# Can we screen for pancreatic cancer? Identifying a sub-population of patients at high risk of subsequent diagnosis using machine learning techniques applied to primary care data

**DOI:** 10.1371/journal.pone.0251876

**Published:** 2021-06-02

**Authors:** Ananya Malhotra, Bernard Rachet, Audrey Bonaventure, Stephen P. Pereira, Laura M. Woods

**Affiliations:** 1 Faculty of Epidemiology and Population Health, Department of Non-Communicable Disease Epidemiology, Inequalities in Cancer Outcomes Network, London School of Hygiene & Tropical Medicine, London, United Kingdom; 2 Epidemiology of Childhood and Adolescent Cancers Team, CRESS, Université de Paris-INSERM, Villejuif, France; 3 UCL Institute for Liver and Digestive Health, University College London, London, United Kingdom; Centro Nacional de Investigaciones Oncologicas, SPAIN

## Abstract

**Background:**

Pancreatic cancer (PC) represents a substantial public health burden. Pancreatic cancer patients have very low survival due to the difficulty of identifying cancers early when the tumour is localised to the site of origin and treatable. Recent progress has been made in identifying biomarkers for PC in the blood and urine, but these cannot be used for population-based screening as this would be prohibitively expensive and potentially harmful.

**Methods:**

We conducted a case-control study using prospectively-collected electronic health records from primary care individually-linked to cancer registrations. Our cases were comprised of 1,139 patients, aged 15–99 years, diagnosed with pancreatic cancer between January 1, 2005 and June 30, 2009. Each case was age-, sex- and diagnosis time-matched to four non-pancreatic (cancer patient) controls. Disease and prescription codes for the 24 months prior to diagnosis were used to identify 57 individual symptoms. Using a machine learning approach, we trained a logistic regression model on 75% of the data to predict patients who later developed PC and tested the model’s performance on the remaining 25%.

**Results:**

We were able to identify 41.3% of patients < = 60 years at ‘high risk’ of developing pancreatic cancer up to 20 months prior to diagnosis with 72.5% sensitivity, 59% specificity and, 66% AUC. 43.2% of patients >60 years were similarly identified at 17 months, with 65% sensitivity, 57% specificity and, 61% AUC. We estimate that combining our algorithm with currently available biomarker tests could result in 30 older and 400 younger patients per cancer being identified as ‘potential patients’, and the earlier diagnosis of around 60% of tumours.

**Conclusion:**

After further work this approach could be applied in the primary care setting and has the potential to be used alongside a non-invasive biomarker test to increase earlier diagnosis. This would result in a greater number of patients surviving this devastating disease.

## Introduction

Patients with pancreatic cancer (PC) are predominantly diagnosed with late stage disease when curative treatment is rarely possible resulting in very low survival and an important public health burden [1–3]. It is probable that years elapse between the initiation of pancreatic cancer and diagnosis but that many patients display only non-specific symptoms during this time [4]. Progress to increase the proportion of cancers diagnosed at an early enough stage has been extremely slow [5,6]. Population-based screening for pancreatic cancer is not a viable option due to the very low incidence (32, 202 and 300/100,000 amongst persons 40–59, 60–79 and 80–99 years respectively) [7,8] resulting in a prohibitively expensive, and even potentially damaging programme [1,9].

**Fig 1:**
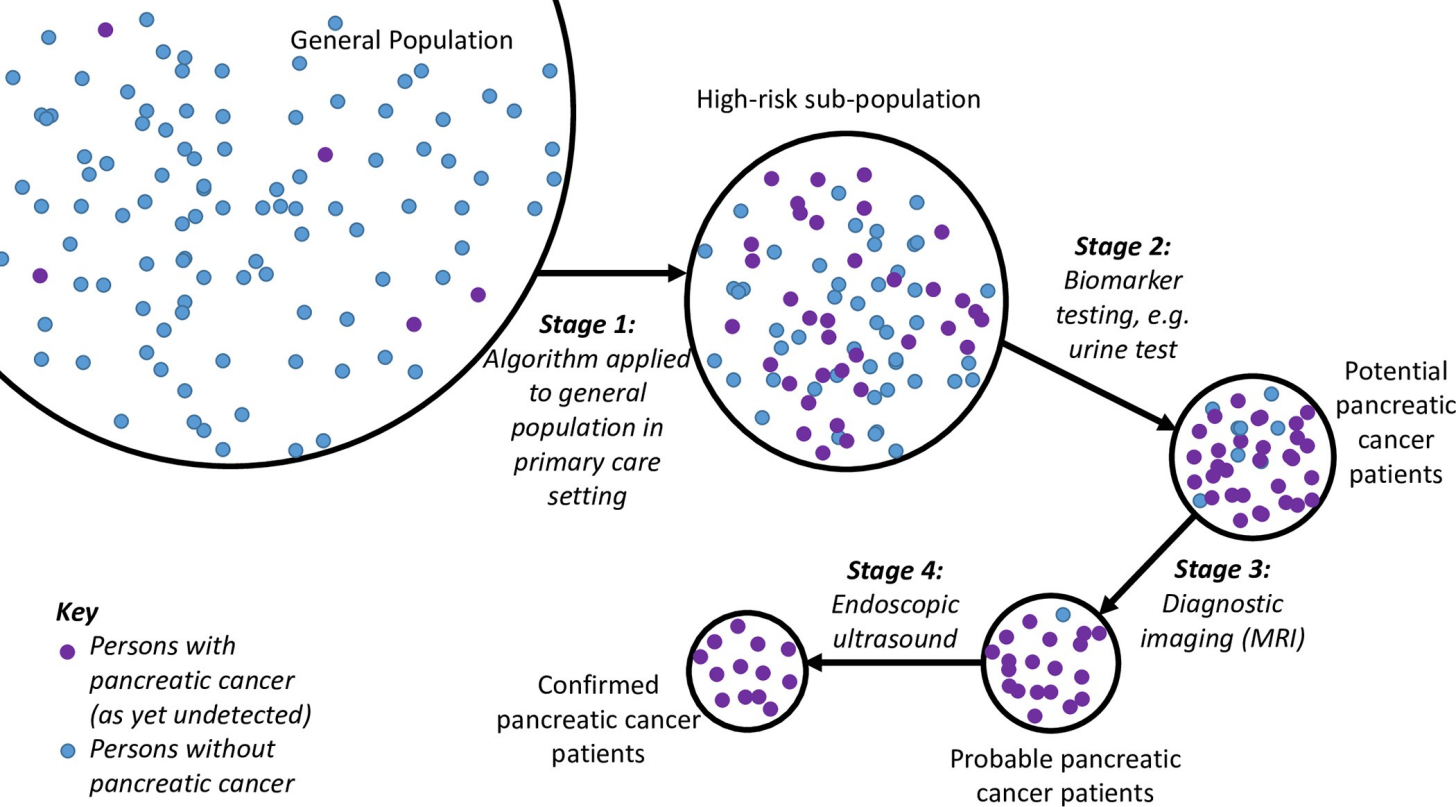
Schematic of multi-stage screening process starting with machine-learning derived algorithm.

Targeted screening, on the other hand, is a possibility. Diagnostic biomarkers [10–14] have been actively sought for a number of years, and the most promising results have been obtained using protein markers [15–17] either alone or in combination with the clinically established biomarker, CA 19–9 [18]. The authors of these molecular studies mostly identify their application to very high-risk groups containing small numbers of people, for example, those with at least two affected first-degree relatives or individuals with known underlying gene abnormalities [19]. However, such tests could be applied more systematically on a suitable high-risk sub-population. Ideally, this high-risk sub-population would be identified within the primary care setting among the general population, as the first step of a multi-stage, targeted screening model (Fig 1).

This research examines the feasibility of identifying such a sub-population using routinely recorded primary health care data. Our hypothesis is that patients who later develop pancreatic tumours share similar profiles in terms of early, diffuse, warning signs which are detectable within electronic health records.

## Materials and methods

### Data

The English National Cancer Registry (CR) was individually linked to Clinical Practice Research Datalink (CPRD). These data were released under national statutory approvals from The Confidentiality Advisory Group (CAG): PIAG 1-05(c)2007, PIAG 3-06(f) 2008 and national ethical approvals from the Research Ethics Committee (REC): 13-LO-0610, 08-H1102-46.

CPRD is a complex, comprehensive database of anonymised electronic health records for individual patients registered at general practices (GP) across the UK. It contains patient demographic information, clinical diagnoses, prescriptions, immunisations, test results and certain information on health and health-seeking behaviours. It is one of the largest sources of continuous patient records available and is broadly representative of patient and practice characteristics in the UK. Research-quality (up-to-standard, UTS) data are available for 13 million patients, five million of whom are currently registered, submitted to the central database by over 600 UK primary care centres. Linkage of CPRD primary care data with other patient level datasets, including cancer registrations, is available for English practices within CPRD who have consented to participate in the linkage scheme.

### Case definition and control selection

Cases were defined as patients aged 15–99 years diagnosed with primary pancreatic cancer (ICD-10 code C25) between January 1, 2005 and June 30, 2009 whilst registered with a CPRD practice who had been submitting UTS data for at least two years before their diagnosis (S1 Fig). Controls were patients diagnosed with an unrelated primary cancer at least 18 months after the index date (date of diagnosis of matched PC case) who were similarly registered within a CPRD practice submitting UTS data during the identical chronological period. We excluded patients diagnosed with cancers of the lip, oral cavity and pharynx (ICD-10 codes C00-14), digestive organs (C15-26), respiratory and intrathoracic organs (C30-39), breast (C50) and, female genital organs (C51-58) which share at least one symptom or risk factor with pancreatic cancer. Each case was matched to four controls on the basis of sex and birth cohort ± 2.5 years.

### Variable coding

We derived symptoms and health statuses of patients known to be associated, or possibly associated with a subsequent diagnosis of pancreatic cancer during the two-year period prior to diagnosis (S1 Table). First, an exhaustive list of medical or product (drug) codes associated with each symptom was derived from the Code Browser using broad search terms. Code lists for frequently studied symptoms and drug use were also obtained via correspondence with authors of published papers as well as from the LSHTM Data Compass [20], a curated digital repository of research outputs produced by staff and students at the London School of Hygiene & Tropical Medicine (LSHTM) and their collaborators. We also searched for clinical codes on CALIBER [21] which is a comprehensive, open access resource for UK electronic health records data.

Medical and drug prescription codes along with their event dates up to two years prior to index date of pancreatic cancer were extracted using Clinical and Therapy files provided by CPRD. We also searched for useful information in the Additional, Referral and Test files. Codes were assigned to an appropriate symptom to build an analysable data structure. These symptoms aimed to be as comprehensive as possible; derived from both extensive literature searches as well as discussions with our clinical collaborator (SPP). After establishing a clean and exhaustive code list for each symptom and drugs prescribed, the codes were checked for non-repetition across all the different variables. Fifty-three symptoms were coded in this fashion.

We derived patients’ status in relation to smoking, body mass index (BMI) [22], diabetes and, alcohol consumption using previously-published code lists. We allocated ‘ever smoker’ status if patients had been recorded as being a current smoker or ex-smoker in the past two years prior to index date, whilst ‘ever heavy drinker’ status was similarly attributed to patients recorded as current or ex heavy alcohol consumers. Any recording of BMI in the previous 24 months (or height and weight on the same day) was extracted. If the most recent BMI prior to cancer diagnosis was greater than or equal to 30, the patient’s status was marked as obese. We allocated diabetic status to a patient if (s)he recorded any of the diabetes associated medical codes or were prescribed anti-diabetic drugs during the time window of interest. In addition to medical codes on weight loss entered by the GP, patients were also coded as having experienced weight loss as a symptom if his/her most recent BMI was either five or more per cent lower than their highest recorded BMI or the first BMI in the two years before cancer diagnosis. We used ecologically-derived measures of income deprivation for each person using quintiles of the income domain score from the English Indices of Multiple Deprivation 2004, 2007 and, 2010 [23]. Deprivation categories were derived from the score temporally closest to each person’s index date on the basis of their residential address. The consultation file was used to calculate the total number of appointments a patient had with a doctor at the GP surgery within the time period under study.

All medical records referring to administration and routine examination codes were excluded since they did not contain any clinical information. Symptoms relating to the eyes, ears, skin and, skeleton were also excluded. Finally, we visually examined all the remaining non-assigned codes occurring more than ten times in the database. These included symptoms of cough, sore throat, dizziness, Polymyalgia rheumatica, gout, venesection and, Salicylate prophylaxis. In discussion with our clinical collaborator (SPP), we excluded most of these codes along with the codes which had fewer than ten clinical events within the whole cohort during the entire study period.

### Ethics

This pilot study was conducted on pre-existing linked data in order to evaluate the feasibility of this approach. These data were released under national statutory approvals from The Confidentiality Advisory Group (CAG): PIAG 1-05(c)2007, PIAG 3-06(f) 2008 and national ethical approvals from the Research Ethics Committee (REC): 13-LO-0610, 08-H1102-46. The study protocol was approved by the London School of Hygiene & Tropical Medicine Ethics Committee on April 25, 2019 (Ref 17053).

### Statistical analyses

We fitted models in the machine learning setting using R software [24]. Our aim was to derive a risk score for developing PC for each patient at time points prior to diagnosis. The outcome was the probability (between zero and one) that a patient would develop the disease, derived as a function of the input variables which included all symptoms, status variables (obesity, smoking, alcohol and, diabetes) age, sex, social deprivation and, the number of consultations in the time window of interest.

Initially, all input variables for the full 24 months before diagnosis were included. The model was then refitted for each monthly interval between 1 and 20 prior to diagnosis, disregarding each time information collected after the assessment time point. For example, the model fitted for month one excluded any information recorded less than one month before diagnosis, but included information between months 2–24; the model for 20 months excluded all information recorded prior to 20 months, but included information for months 21–24. Multivariate logistic regression (LR) and random forest (RF) models were fitted within the supervised machine learning setting. We regarded the four patient statuses as non-time varying by recording them as binary (yes/no) variables if they occurred within the time window of interest. Symptoms were fitted as continuous variables by including the count of the number of times a symptom or drug was reported during the time window of interest.

The models were trained on 75% of the data (the training set) and then assessed on the remaining 25% (the test set) by the derivation of the area under the receiver operating characteristic curve (AUC), sensitivity, and specificity. Cases and their matched controls were kept together in one or other of the sets. The model’s performance measures (AUC, sensitivity and specificity) were assessed at different threshold values (cut-off points) between 0.1 to 0.5. If the predicted probability of a patient’s outcome was higher than the threshold value, then they were considered ‘high-risk’ of PC. The analyses were conducted by age in order to compare younger patients (aged 60 years and under) to older patients (more than 60 years).

## Results

### Descriptive analyses

We identified 1,144 patients with primary pancreatic tumours eligible for inclusion. Four age- and sex- matched controls were identified for 1,139 cases, whose male-to-female ratio was 0.957 and whose median age at diagnosis was 71 years. We were unable to find four controls for the remaining five cases: these were females with mean age of 93 years all of whom died within seven months from diagnosis. These cases were excluded.

We derived the percentage of cases and controls who reported each symptom at any time during the 24 months prior to pancreatic cancer diagnosis (cases) or the index date (controls) (Table 1). Pearson’s chi-square test [25] was used to test if these observed differences between cases and controls for each symptom were by chance. Abdominal pain, irritable bowel syndrome and, constipation symptoms were reported around four times more frequently among cases than controls while gastrointestinal conditions and diabetes were twice as common. There was a negligible difference in the prevalence of genitourinary and immunological disorders. The prevalence of each symptom in every monthly interval before diagnosis among cases and controls varied by symptom. For example, reporting of jaundice was very different for cases and controls but only very close to diagnosis (S2–S9 Figs). By contrast, higher prevalence of gastrointestinal problems amongst cases was evident from 24 months onwards.

**Table 1:** Percentage of cases and controls reporting symptoms or statuses within the 24-month period prior to diagnosis.

	Cases [%] (*n* = 1139)	Controls [%] (*n* = 4556)	p-value
***Cardiovascular disorders***			
Cardiovascular diseases	279 [24.5%]	758 [16.64%]	<0.0001
Hypertension	216 [18.96%]	798 [17.52%]	0.9693
***Circulatory system disorders***			
Migratory thrombophlebitis	0 [0%]	0 [0%]	NA
***Digestive disorders***			
Abdominal pain	368 [32.31%]	319 [7%]	<0.0001
Jaundice	251 [22.04%]	11 [0.24%]	<0.0001
Gastrointestinal conditions	350 [30.73%]	547 [12.01%]	<0.0001
Constipation	134 [11.76%]	119 [2.61%]	<0.0001
Oesophago-gastric problems	67 [5.88%]	109 [2.39%]	<0.0001
Irritable bowel syndrome	53 [4.65%]	58 [1.27%]	<0.0001
Diverticular disease	36 [3.16%]	63 [1.38%]	0.0004
Pancreatitis	15 [1.32%]	2 [0.04%]	<0.0001
Abdominal mass	12 [1.05%]	2 [0.04%]	<0.0001
Odynophagia	15 [1.32%]	14 [0.31%]	0.0002
Gallbladder diseases	17 [1.49%]	26 [0.57%]	0.0064
Flatulence	10 [0.88%]	9 [0.2%]	0.0023
Oral problems	10 [0.88%]	12 [0.26%]	0.0125
Xerostomia	6 [0.53%]	12 [0.26%]	0.3423
Inflammatory bowel disease	4 [0.35%]	5 [0.11%]	0.2002
Stomatitis	2 [0.18%]	11 [0.24%]	0.8489
Halitosis	1 [0.09%]	2 [0.04%]	1
Glossodynia	0 [0%]	0 [0%]	NA
Steatorrhoea	0 [0%]	0 [0%]	NA
***Diseases of the musculoskeletal system and connective tissue***			
Back pain	191 [16.77%]	377 [8.27%]	<0.0001
Rheumatoid arthritis	6 [0.53%]	14 [0.31%]	0.5046
Pruritis	33 [2.9%]	63 [1.38%]	0.0026
***Drugs***			
On Opioids	281 [24.67%]	327 [7.18%]	<0.0001
On Antiplatelets	333 [29.24%]	890 [19.53%]	<0.0001
On HRT	116 [10.18%]	174 [3.82%]	<0.0001
On NSAIDS	293 [25.72%]	919 [20.17%]	0.0063
***Endocrine and Metabolic disorders***			
Diabetes	270 [23.71%]	439 [9.64%]	<0.0001
Polydipsia	5 [0.44%]	5 [0.11%]	0.0684
Hyperlipidaemia	47 [4.13%]	167 [3.67%]	0.9027
***Genitourinary disorders***			
Kidney problems	80 [7.02%]	237 [5.2%]	0.0969
Urinary problems	107 [9.39%]	356 [7.81%]	0.3727
Gynaecological conditions	29 [2.55%]	95 [2.09%]	0.6616
Endometriosis	0 [0%]	4 [0.09%]	0.6643
Fibroids	1 [0.09%]	2 [0.04%]	1
Dysmenorrhoea	0 [0%]	0 [0%]	NA
***Haematological disorders***			
Anaemia	50 [4.39%]	83 [1.82%]	<0.0001
***Immunological disorders***			
Atopic diseases	147 [12.91%]	489 [10.73%]	0.2757
Auto-immune diseases	34 [2.99%]	126 [2.77%]	1
***Infections***			
Mumps	1 [0.09%]	0 [0%]	0.4854
***Nervous system***			
Insomnia	48 [4.21%]	93 [2.04%]	0.0003
Hypersomnia	0 [0%]	0 [0%]	NA
***Oncological disorders***			
Family history of breast cancer	1 [0.09%]	3 [0.07%]	1
Peutz-Jeghers syndrome	0 [0%]	0 [0%]	NA
Familial atypical multiple mole melanoma (FAMMM)	0 [0%]	0 [0%]	NA
***General clinical symptoms***			
Weight loss	380 [33.36%]	437 [9.59%]	<0.0001
Fatigue/Malaise	102 [8.96%]	145 [3.18%]	<0.0001
Anorexia	52 [4.57%]	8 [0.18%]	<0.0001
Anxiety/Depression	115 [10.1%]	246 [5.4%]	<0.0001
Weakness	23 [2.02%]	34 [0.75%]	0.0008
Fever	15 [1.32%]	41 [0.9%]	0.4088
Obesity	171 [15.01%]	465 [10.21%]	0.0003
Disturbances in smell/taste	0 [0%]	5 [0.11%]	0.5342
***Health behaviours***			
Ever smoker	474 [41.62%]	1275 [27.99%]	<0.0001
Ever heavy drinker	11 [0.97%]	25 [0.55%]	0.2535

Month by month, cases reported more symptoms than controls throughout the whole period of observation (S10 Fig). The cumulative median number of symptoms in each time window was higher among cases but still substantial among controls (S10 Fig). The number of symptoms rose towards the diagnosis in both groups but especially in cases. Amongst patients reporting at least one symptom in a given month (on average, 80% of cases and 70% of controls) cases reported much higher total number of symptoms than controls in the same period (S10 Fig).

### Multivariable regression

Multivariate logistic regression (LR) and random forest (RF) models presented similar results, indicating that the choice of model was not important. For simplicity we henceforth report outputs from only logistic regression models.

**Fig 2:**
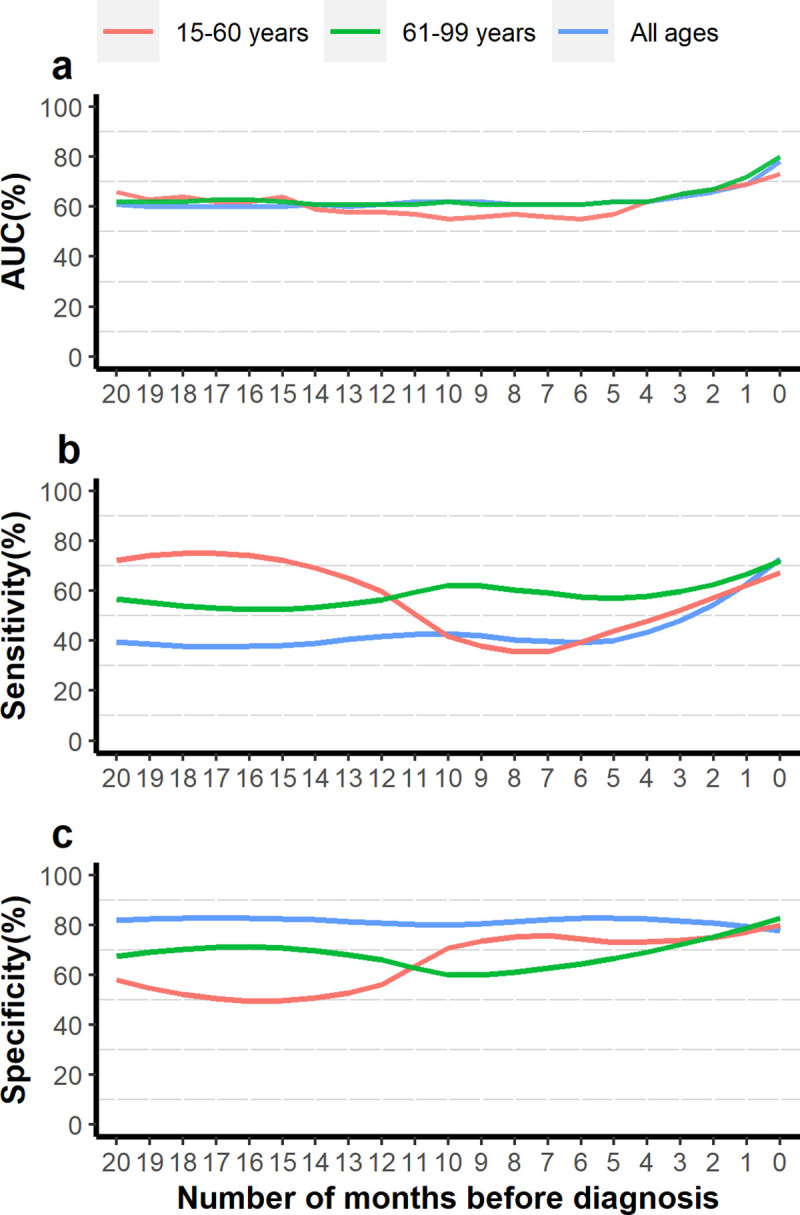
Model parameters obtained in each time window (0–24,1–24,…,20–24 months) prior to diagnosis. a) AUC (%) b) Sensitivity (%) c) Specificity (%).

The performance of the models was checked in the 25% test set in every time window up to 20–24 months preceding diagnosis. An optimal cut-off for the predicted probability of outcome, where AUC is maximal, was determined separately for models in each time window. The AUC, sensitivity and, specificity were plotted corresponding to these optimal cut-offs against varying time periods. The AUC (which represents a measure or degree of separability of a true positive from a false positive at various threshold values) was optimal (highest) when using all available 24 months, including the period immediately prior to diagnosis, and decreased as the time window of analysis moved further away from diagnosis (Fig 2A). Sensitivity (the ability to correctly detect a patient who developed pancreatic cancer) was maximized at 73% at the time of diagnosis, dropping to 35% in younger and 53% in older patients in time windows prior to 6 months from diagnosis (Fig 2B). Specificity (the ability to correctly detect a patient who did not develop PC) ranged from 75%-88% across all time windows for all patients (Fig 2C). The model’s performance varied by age, with higher sensitivity and lower specificity in earlier months in the age-specific analyses, whilst the AUC was similar.

**Fig 3:**
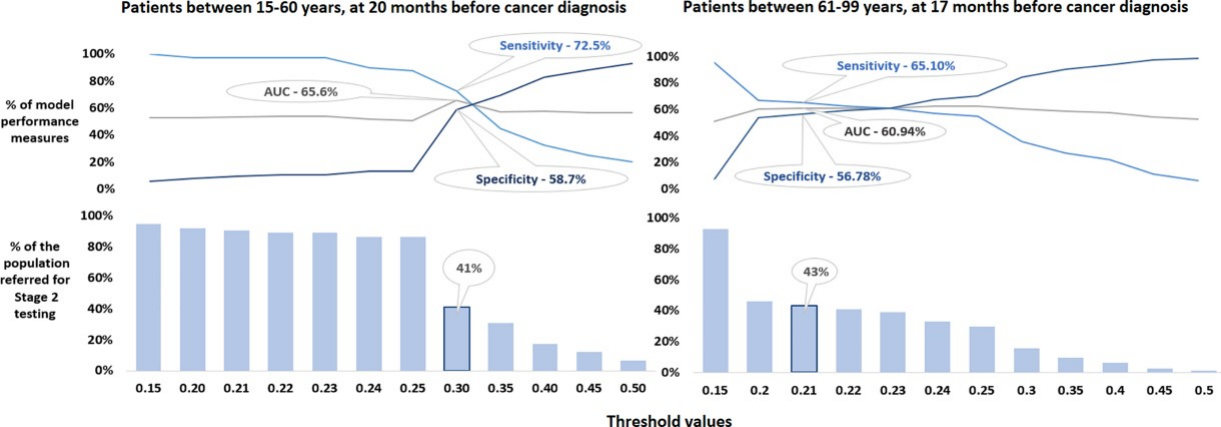
AUC, sensitivity and specificity of the models with % of the population recommended for biomarker (Stage 2) testing.

Since we were principally interested in maximizing sensitivity early in the course of the disease, further analyses were conducted on age-specific models for the time window more than 12 months prior to diagnosis which displayed the maximal AUC: 20 months for younger patients and 17 months for older patients (S2–S5 Tables). For each of these time windows we plotted age-specific model’s performance measures against various values in order to establish the optimal cut-off point. For patients aged 60 or under, this was 0.3 (Fig 3) where the AUC was 65.6%, the sensitivity of this model was 72.5% and, the specificity was 58.7%. In the population of patients over 60 years, the optimal cut-off was 0.21 where the AUC was 60.9%, the sensitivity was 65.1% and, specificity of 56.8% (Fig 3).

Our algorithm identified 41.3% (Fig 3) of younger patients and 43.2% (Fig 3) patients being classified into a ‘high-risk sub-population’ (Fig 1). Using the most recently published incidence for England [7,8] this equates to 10,923 persons per pancreatic cancer present in younger patients and 740 in older patients. Were a biomarker with a sensitivity of 87.5% [17] applied, around 30 older and 400 younger persons would be identified as ‘potential pancreatic cancer patients’ (those referred on for imaging and potentially endoscopy) leading to the early detection of around 63% of pancreatic cancers in younger patients and 57% in older patients. (Table 2).

**Table 2:** Parameters of the optimal logistic regression models envisioned in the context of the hypothetical multi-stage screening model*: England 2017.

		Age-group (years)	
		15–60	61–99
		Median age (5^th^, 95^th^) percentile	
		55 (42, 59)	76 (62, 92)
**Incidence rate [7,8]**	*A*	0.00003781	0.00058378
**Tumours registered per 100,000 persons**		3.8	58.4
**Optimal model time window (months)**		20–24	17–24
**AUC**		65.6%	60.9%
**Sensitivity**	*B*	72.5%	65.1%
**Specificity**	*C*	58.7%	56.8%
**Positive predictive value**		40.0%	32.5%
**Negative predictive value**		72.2%	83.6%
**Proportion identified as ’high-risk’**	*D = (A×B) + ((1-A)×(1-C))*	0.41	0.43
**Persons identified as ’high-risk’ per cancer present**	*E = D/A*	10,923	740
**Best estimate of biomarker sensitivity [17]**	*F*	87.5%	87.5%
**Best estimate of biomarker specificity [17]**	*G*	96.4%	96.4%
Persons referred for Stage 2* testing per cancer present	*H = (1×F) + ((E-1)×(1-G)*	394	27
**Maximum proportion of cancers detected**	*J = B×F*	0.63	0.57

*See Fig 1.

Diabetes was the principal influential risk factor in younger patients. (S2 and S4 Tables). Since we learned the importance of diabetes in both age groups, we observed 89% of the younger and 96% of the older diabetic patients correctly predicted to develop PC, as opposed to 68% of the younger and 47% of the older non-diabetics.

## Discussion

We have demonstrated that it is possible to detect a sub-population of people at higher risk of developing pancreatic cancer in the primary care setting up to two years before the cancer is diagnosed. Our algorithm scales down the general population potentially at high risk of this disease by more than a half, and suggests that it could be possible to diagnose around 60% of pancreatic cancers early. Although the total numbers of persons who would need to be tested, particularly in younger patients, is unfeasibly high, this is likely to be largely driven by the use of cancer patients as controls. Our study thus suggests that logistic regression has potential within the machine learning setting to enable a multi-stage screening model for pancreatic cancer, but does not yet demonstrate high enough specificity to be applied in practice without further calibration and development.

### Public health context

It is widely accepted that pancreatic cancer is not a good candidate for mass population-based screening due to its low incidence in the general population. As small as 10% of the patients diagnosed with pancreatic ductal adenocarcinoma (PDAC) have either a strong family history of PC, hereditary pancreatitis or a particular genetic syndrome [1]. The remaining 90% of PDAC patients are non-inherited cases who overwhelmingly display no alarm symptoms until the tumour is well-advanced.

### Strengths and limitations

This study has a number of strengths. First, we used prospectively collected data and a high-quality database. Under-recording or inaccurate reporting may be present but is unlikely to be differential. We evaluated temporal patterns by establishing the predictive accuracy of our model at separate time points throughout the two-year period leading up to diagnosis. We used the machine learning setting to carry out statistical analyses, rather than multivariable regression methods or conditional logistic regression as has previously been implemented [26,27]. Machine learning techniques are more powerful in settings such as this one where they are more likely to identify numerous weak signals which are only predictive when used in complex combination with each other. Both of the prediction models (LR and RF) produced similar outcomes suggesting that our results are valid irrespective of the model choice. This is an advantage of applying machine learning techniques in our study setting which confirms comparable predictions using two different regression models.

The one major limitation of our study is the poor specificity of our models arising principally from the use of cancer patients as controls. Our controls were selected from cancer patients diagnosed later in time with unrelated malignancies. Despite the fact that we ensured that the controls’ own diagnosis was at least 18 months after that of their matched case, they nevertheless reported increasing numbers of symptoms of interest over the study period in a fairly similar fashion to that of the cases. This implies that the controls are not representative of the general population, and explains why our models predict a high number of false positives. However, in the light of this limitation, our results are still promising: the models achieve a relatively high sensitivity and the specificities of the final models are in excess of 55%. Repeating the analyses using full population-based controls would lead to a much higher specificity and is the first priority in progressing this research.

Our results suggest that further age differentiation is required to truly evaluate the utility of this approach in patients under 60 years, which would be possible in a larger dataset. In particular, it would be important to evaluate performance separately for patients aged 40–49 and 50–59 amongst whom the potential effectiveness of our models is currently obscured by the very low incidence amongst 15-59-year olds. Initial calculations show that if the model parameters for ages 15–59 held for the limited age group of 40–59 years (median age 55 years) 1,290 persons per pancreatic cancer would have been identified as ‘high-risk’ and 47 as ‘potential cancer patients’ (compared to 10,923 and 394 for the age group 15–59).

Further limitations are relatively minor. Alcohol consumption and smoking habits are almost certainly under-reported in primary care [28,29], but arguably accurately reflect what is known to the GP at any given time and thus are as good as clinical knowledge of any other symptom. Patient status was recorded as ‘diabetic’ if this disease was reported in the time window of interest which did not permit us to distinguish between long-term or recent onset disease.

### Comparison with existing literature

Previous authors have highlighted putative features associated with PC diagnosis such as jaundice, abdominal pain, back pain, gastric problems, weight loss, malaise and new-onset diabetes [26,30]. Whilst our results are consistent with these findings, we have examined whether it is possible to predict future PC diagnosis based on the presence or absence of symptoms or abnormalities present more than 12 months before diagnosis, ignoring late stage symptoms. Comparison with existing literature is, therefore, limited.

### Implications for future research

This study is preliminary, and thus needs to be expanded and extended in order for these findings to be confirmed. First, it is essential that future analyses use population-based controls so that true specificity of the algorithm within a population-based database can be derived. Second, analyses should differentiate between diabetics and non-diabetics, as well as in a more refined manner by age. A notable result was the relative importance of diabetes, over time-varying symptoms, in predicting later pancreatic cancer diagnosis, which is consistent with previous research [31]. Since studies have established a particularly increased risk amongst new onset diabetics [1], stratified analyses by diabetic history hold promise to improve the predictive ability of the model for all patients. Third, testing the algorithm in ‘real time’, where a primary care database is interrogated as if this algorithm were in use is important to understand its cost-effectiveness. In reality, the greatest potential of this algorithm lies within a multiple-testing model, where a number of different biomarkers for several different malignancies are evaluated simultaneously within the same patient. This study is an important first step to establishing such a programme.

### Conclusion

We have demonstrated that it is possible to discriminate a high-risk sub-population of patients whom are more likely to go on to be diagnosed with pancreatic cancer from routine primary care records. Our results could lead to increasing early diagnosis through a multi-stage screening model which utilises recently developed biomarkers applied to the ‘high-risk’ population identified using this approach. Further work is certainly required to confirm, refine and evaluate the potential use of these findings in practice.

## Supporting information

S1 Fig Criteria for case selection and control matching. (TIF)

S2 Fig Prevalence(%) of cardiovascular disorders in each month before diagnosis among cases and controls. (TIF)

S3 Fig Prevalence(%) of digestive disorders in each month before diagnosis among cases and controls. (TIF)

S4 Fig Prevalence(%) of diseases of the musculoskeletal system and connective tissue in each month before diagnosis among cases and controls. (TIF)

S5 Fig Prevalence(%) of endocrine and metabolic disorders in each month before diagnosis among cases and controls. (TIF)

S6 Fig Prevalence(%) of genitourinary disorders in each month before diagnosis among cases and controls. (TIF)

S7 Fig Prevalence(%) of haematological disorders in each month before diagnosis among cases and controls. (TIF)

S8 Fig Prevalence(%) of auto-immune diseases in each month before diagnosis among cases and controls. (TIF)

S9 Fig Prevalence(%) of general clinical symptoms in each month before diagnosis among cases and controls. (TIF)

S10 Fig Symptoms reported by cases and controls in each time window. a) Median in each month b) Cumulative median c) Percentage of patients.
(TIF)

S1 Table List of variables. (DOCX)

S2 Table Multivariate logistic regression model fitted at month 20 before diagnosis for age-group up to 60 years. (DOCX)

S3 Table Multivariate logistic regression model fitted at month 17 before diagnosis for age-group above 60 years. (DOCX)

S4 Table Random forest model fitted at month 20 before diagnosis for age-group up to 60 years. (DOCX)

S5 Table Random forest model fitted at month 17 before diagnosis for age-group above 60 years. (DOCX)
