# Supplementary figures and images for: Can we screen for pancreatic cancer? Identifying a sub-population of patients at high risk of subsequent diagnosis using machine learning techniques applied to primary care data
Source: PLoS One. 2021 Jun 2;16(6):e0251876. doi: 10.1371/journal.pone.0251876 (PMC8171946; doi:10.1371/journal.pone.0251876)

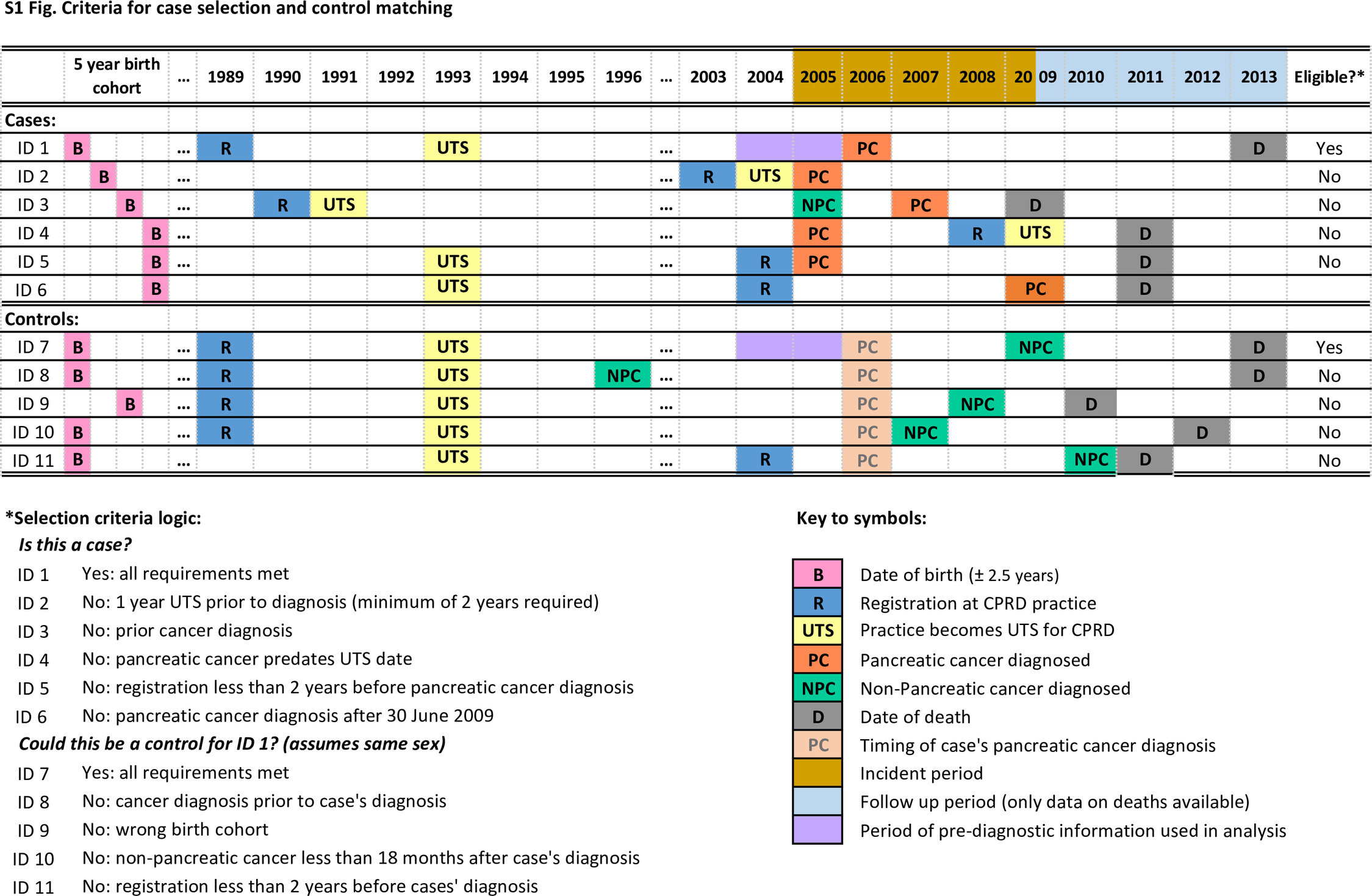

Supplement: S1 Fig — (TIF) [file pone.0251876.s001.tif]

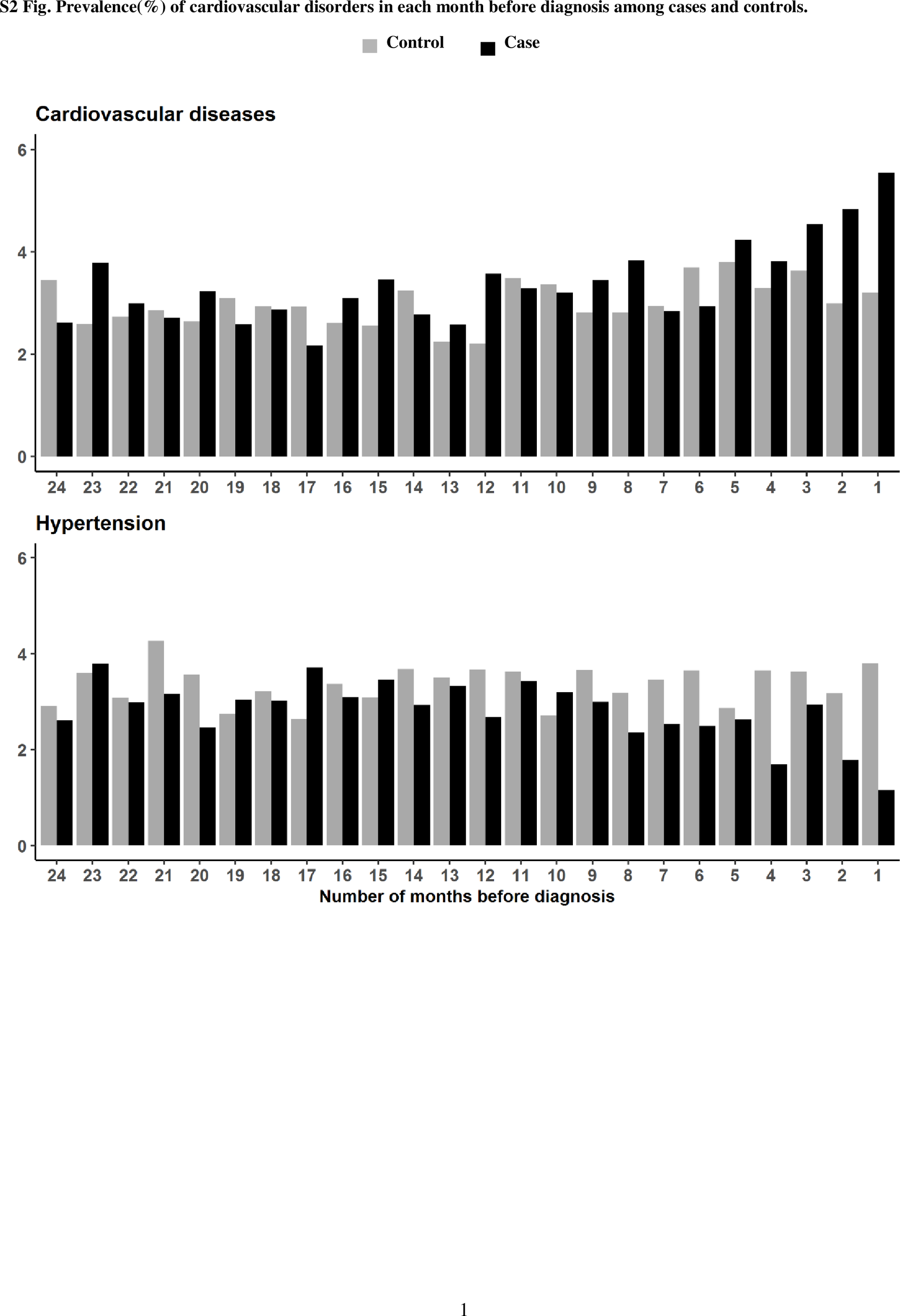

Supplement: S2 Fig — (TIF) [file pone.0251876.s002.tif]

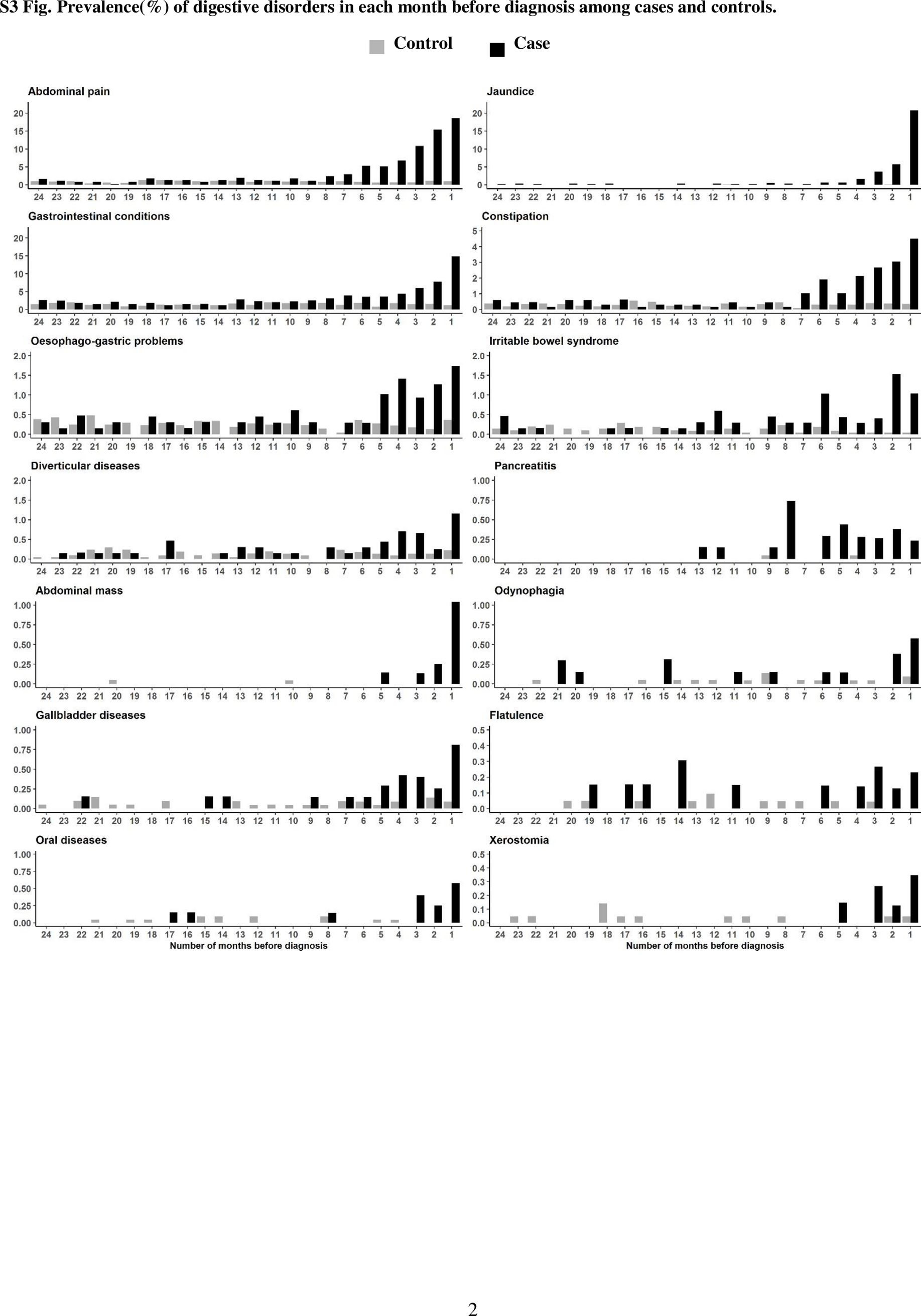

Supplement: S3 Fig — (TIF) [file pone.0251876.s003.tif]

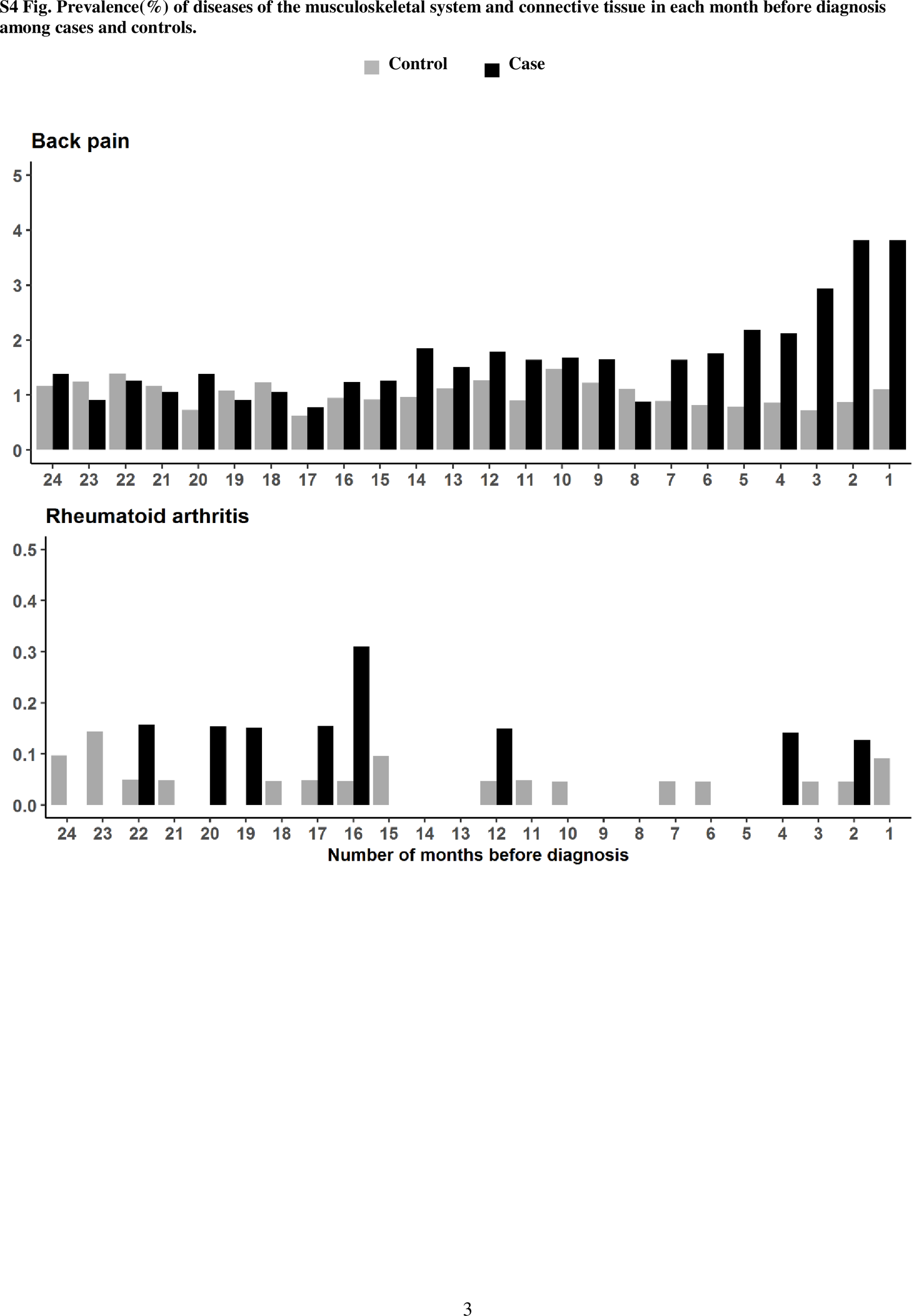

Supplement: S4 Fig — (TIF) [file pone.0251876.s004.tif]

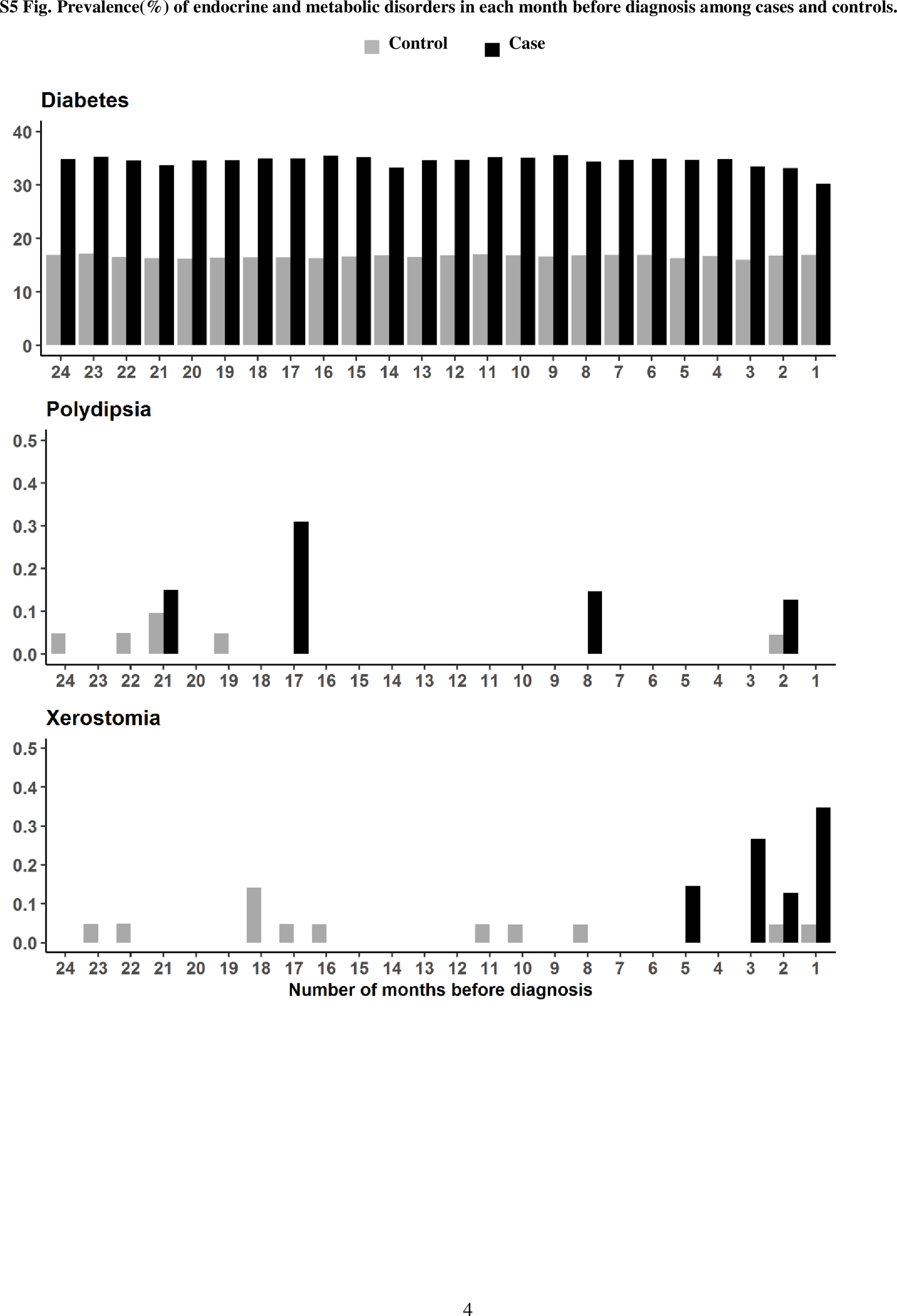

Supplement: S5 Fig — (TIF) [file pone.0251876.s005.tif]

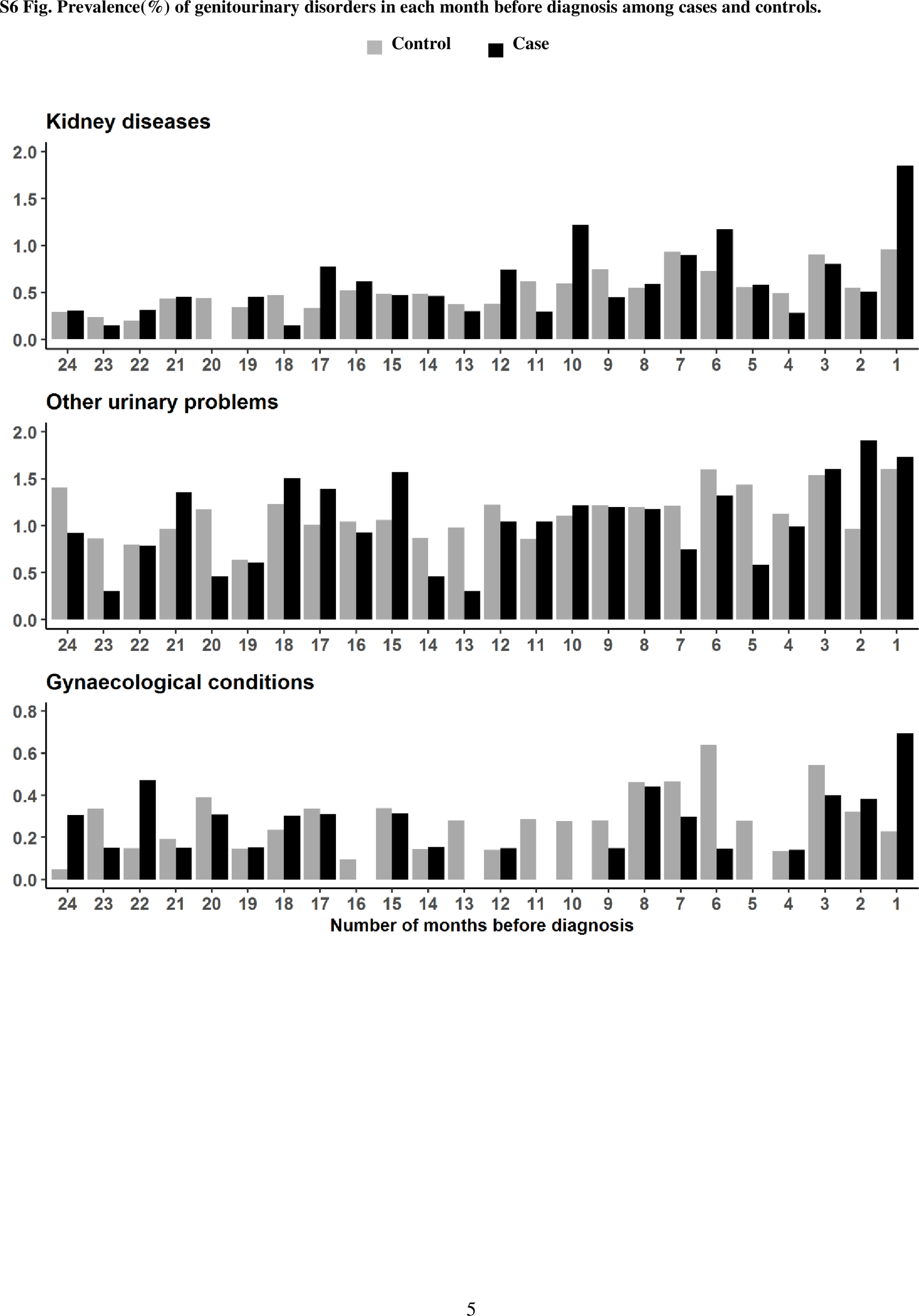

Supplement: S6 Fig — (TIF) [file pone.0251876.s006.tif]

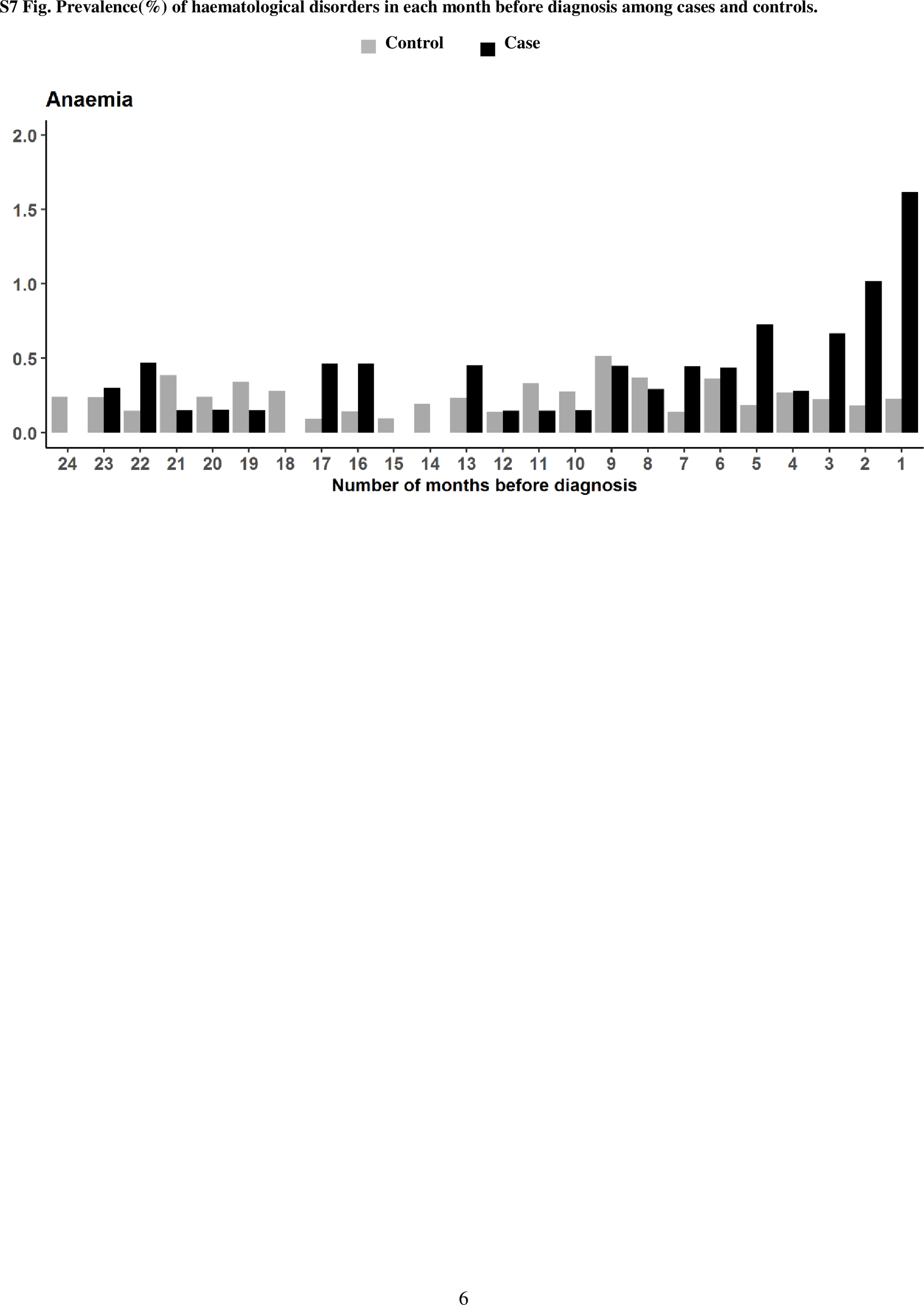

Supplement: S7 Fig — (TIF) [file pone.0251876.s007.tif]

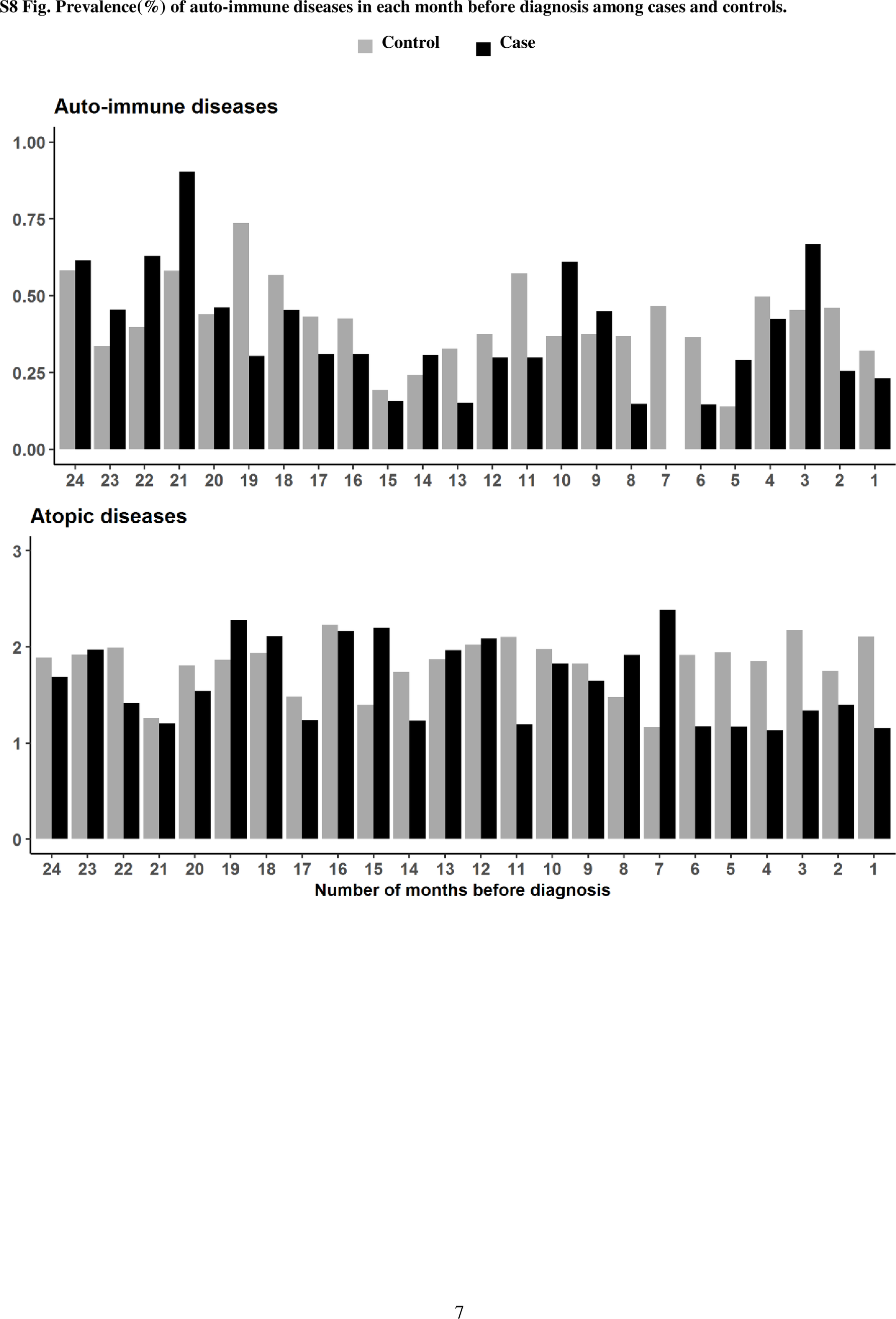

Supplement: S8 Fig — (TIF) [file pone.0251876.s008.tif]

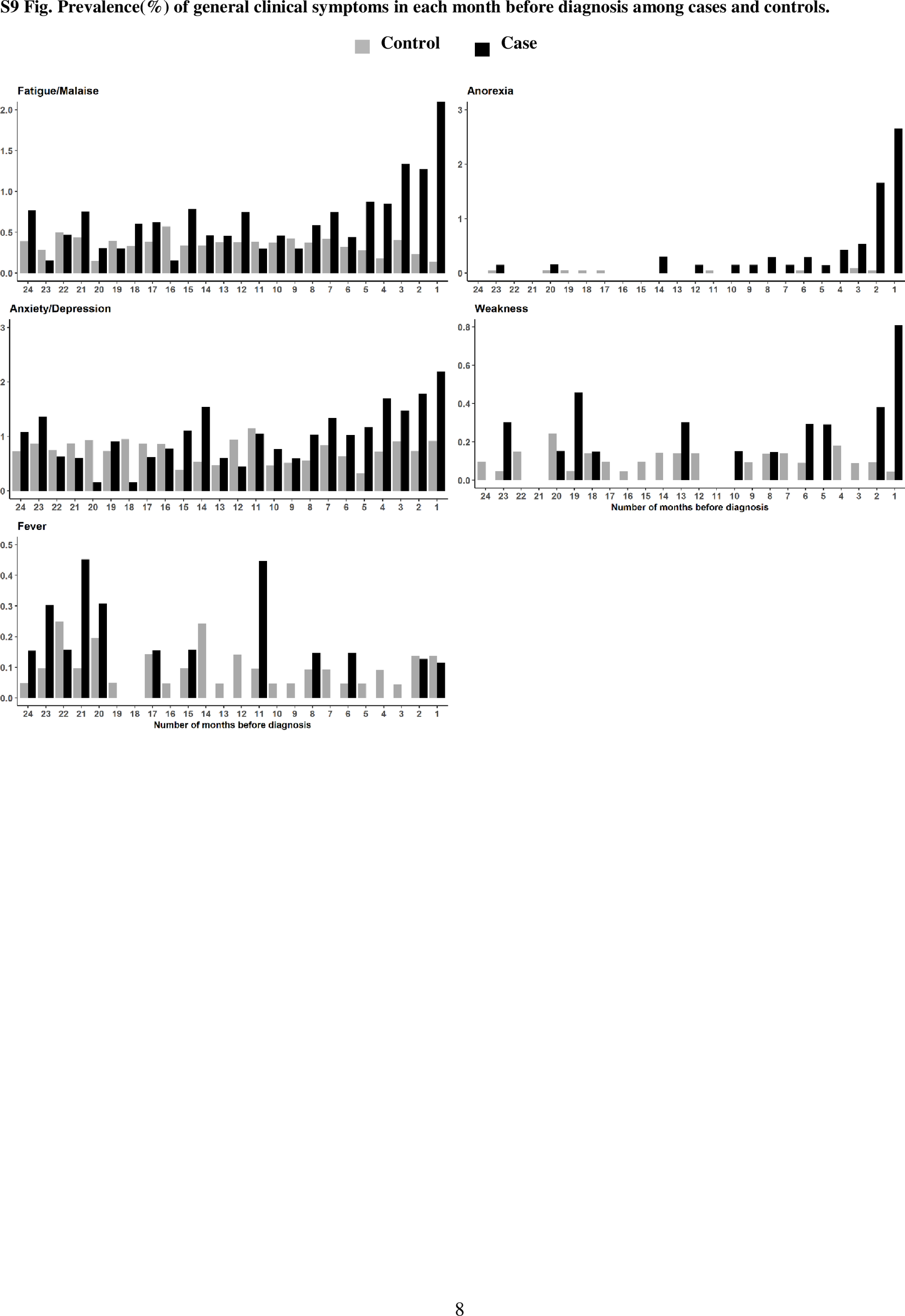

Supplement: S9 Fig — (TIF) [file pone.0251876.s009.tif]

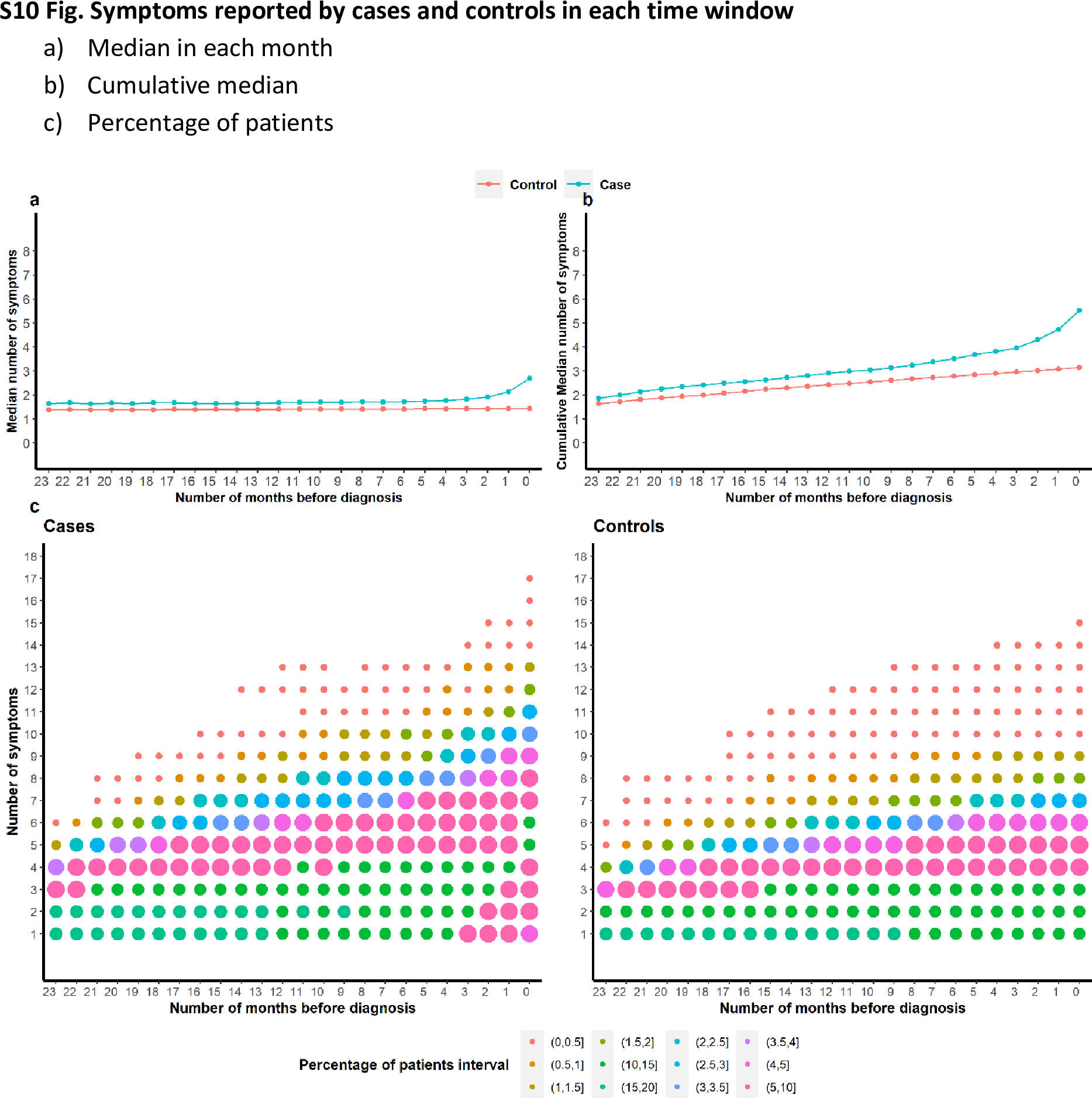

Supplement: S10 Fig — a) Median in each month b) Cumulative median c) Percentage of patients. (TIF) [file pone.0251876.s010.tif]
