# Supplementary material for: Can we screen for pancreatic cancer? Identifying a sub-population of patients at high risk of subsequent diagnosis using machine learning techniques applied to primary care data
Source: PLoS One. 2021 Jun 2;16(6):e0251876. doi: 10.1371/journal.pone.0251876 (PMC8171946; doi:10.1371/journal.pone.0251876)
Supplement: S1 Table — (DOCX) [file pone.0251876.s011.docx]

**S1 Table. List of variables**

| **Symptoms and Statuses** | **No. of Read Codes** | **Search term in CPRD Code** | **Existing code list link** | | **Existing Literature** | | |
| --- | --- | --- | --- | --- | --- | --- | --- |
|  |  |  |  |  | **Paper** | **Year** | **URL** |
| Smoking | 157 |  |  | |  |  |  |
| Alcohol | 184 |  |  | |  |  |  |
| BMI | calculated using weight  and height recorded on the same day |  |  | | Representativeness and optimal  use of body mass index (BMI) in the UK Clinical Practice Research Datalink (CPRD) | 2013 | [https://bmjopen.bmj.com/content/bmjopen/3/9/e003389.full.pdf?with-](https://bmjopen.bmj.com/content/bmjopen/3/9/e003389.full.pdf?with-ds=yes) [ds=yes](https://bmjopen.bmj.com/content/bmjopen/3/9/e003389.full.pdf?with-ds=yes) |
| Weight loss | 9 | *loss* *weight*,  *weight* *loss*, *weigh*,  *reduc* *weigh*, *low* |  | | Recent Progress in Pancreatic  Cancer | 2013 | <https://www.ncbi.nlm.nih.gov/pmc/articles/PMC3769458/> |
|  |  |  |  | | Exocrine pancreatic cancer: symptoms at presentation and their  relation to tumour site and stage | 2005 | [https://www.ncbi.nlm.nih.gov/pub](https://www.ncbi.nlm.nih.gov/pubmed/15960930/) [med/15960930/](https://www.ncbi.nlm.nih.gov/pubmed/15960930/) |
|  |  |  |  | | The risk of pancreatic cancer in symptomatic patients in primary care: a large case-control study  using electronic records | 2012 | [https://www.ncbi.nlm.nih.gov/pub](https://www.ncbi.nlm.nih.gov/pubmed/22617126) [med/22617126](https://www.ncbi.nlm.nih.gov/pubmed/22617126) |
|  |  |  |  | | Signs and symptoms of pancreatic cancer: a population-based case- control study in the San Francisco  Bay area. | 2016 | [https://www.ncbi.nlm.nih.gov/pub](https://www.ncbi.nlm.nih.gov/pubmed/15181621/) [med/15181621/](https://www.ncbi.nlm.nih.gov/pubmed/15181621/) |
| Obesity | 58 | *obese*, *obesi* |  | |  |  |  |
| Fatigue and Malaise | 25 | *fatigue*, *tired*, *tire*,  *asthenia*, *faint* |  | | Signs and symptoms of pancreatic  cancer: a population-based case- control study in the San Francisco Bay area. | 2016 | <https://www.ncbi.nlm.nih.gov/pubmed/15181621/> |
|  | 6 | *malaise* |  | | The risk of pancreatic cancer in symptomatic patients in primary care: a large case-control study  using electronic records | 2012 | <https://www.ncbi.nlm.nih.gov/pubmed/22617126> |
| Anorexia | 16 | *anorex*, *eating*  *disorder*, *eat* *dis*,  *appetite* |  | | Exocrine pancreatic cancer:  symptoms at presentation and their relation to tumour site and stage | 2005 | <https://www.ncbi.nlm.nih.gov/pubmed/15960930/> |
| Anxiety/Depression | 130 | *depression*, *low  mood*, *depressive*,  *antidepressant*, |  | | Recent Progress in Pancreatic  Cancer | 2013 | <https://www.ncbi.nlm.nih.gov/pmc/articles/PMC3769458/> |
|  | 34 | *anxiety* |  | |  |  |  |
| Weakness | 15 | *weakness*, *weak* |  | | Exocrine pancreatic cancer:  symptoms at presentation and their relation to tumour site and stage | 2005 | <https://www.ncbi.nlm.nih.gov/pubmed/15960930/> |
| Fever | 143 | *fever* |  |  | |  |  |
| Disturbances of smell and taste/  Anosmia | 5 | *distur* *smell*, *distur*  *tast*,*anosmi* |  |  | |  |  |
| Family history of pancreatic cancer | 0 | *FH* *pan*, *FH* |  | Incidence of Pathogenic Variants  in Those With a Family History of Pancreatic Cancer | | 2018 | [https://www.ncbi.nlm.nih.gov/pmc/articles/PMC6110858/](https://www.ncbi.nlm.nih.gov/pubmed/15960930/) |
|  |  |  |  | Recent Progress in Pancreatic  Cancer | | 2013 | [https://www.ncbi.nlm.nih.gov/pmc/articles/PMC3769458/](https://www.ncbi.nlm.nih.gov/pubmed/15960930/) |
|  |  |  |  | Risk Factors for Early-Onset and Very-Early-Onset Pancreatic Adenocarcinoma | | 2016 | [https://journals.lww.com/pancreasjournal/Abstract/2016/02000/Risk_Factors_for_Early_Onset_and_Very_Early_Onset.23.aspx](https://www.ncbi.nlm.nih.gov/pubmed/15960930/) |
|  |  |  |  | An Absolute Risk Model to Identify Individuals at Elevated Risk for Pancreatic Cancer in the  General Population | | 2013 | [https://www.ncbi.nlm.nih.gov/pmc/articles/PMC3772857/#](https://www.ncbi.nlm.nih.gov/pubmed/15960930/) |
|  |  |  |  | Risk Factors for Pancreatic  Cancer: Case-Control Study | | 2007 | [https://www.ncbi.nlm.nih.gov/pmc/articles/PMC2423805/](https://www.ncbi.nlm.nih.gov/pubmed/15960930/) |
| Familial Atypical multiple mole  melanoma (FAMMM) | 1 | *mole* *melanoma*,  *mole*, *multiple*  *mole*, *familial*  *mole*, *FAMMM*, |  | American Cancer Society | |  | [https://www.cancer.org/cancer/pancreatic-cancer/causes-risks-](https://www.ncbi.nlm.nih.gov/pubmed/15960930/) [prevention/risk-factors.html](https://www.ncbi.nlm.nih.gov/pubmed/15960930/) |
| Lynch syndrome | 0 | *lynch*, *HNPCC*,  *colorectal cancer*, *non- polyposis*, *hereditary cancer syndromes*, |  | The Risk of Pancreatic Cancer in  Families with Lynch Syndrome | | 2014 | [https://www.ncbi.nlm.nih.gov/pmc/articles/PMC4091624/](https://www.ncbi.nlm.nih.gov/pubmed/15960930/) |
|  |  |  |  | Recent Progress in Pancreatic  Cancer | | 2013 | [https://www.ncbi.nlm.nih.gov/pmc/articles/PMC3769458/](https://www.ncbi.nlm.nih.gov/pubmed/15960930/) |
| Peutz-Jeghers syndrome | 1 | *jegh* |  | Recent Progress in Pancreatic  Cancer | | 2013 | [https://www.ncbi.nlm.nih.gov/pmc/articles/PMC3769458/](https://www.ncbi.nlm.nih.gov/pubmed/15960930/) |
|  |  |  |  | Pancreatic cancer risk in Peutz- Jeghers syndrome patients: a large cohort study and implications for  surveillance. | | 2013 | [https://www.ncbi.nlm.nih.gov/pubmed/23240097/](https://www.ncbi.nlm.nih.gov/pubmed/15960930/) |
|  |  |  |  | Very high risk of cancer in familial  Peutz-Jeghers syndrome. | | 2000 | [https://www.ncbi.nlm.nih.gov/pubmed/11113065/](https://www.ncbi.nlm.nih.gov/pubmed/15960930/) |
| Pancreatitis | 23 | *pancreatitis* |  | Recent Progress in Pancreatic  Cancer | | 2013 | [https://www.ncbi.nlm.nih.gov/pmc/articles/PMC3769458/](https://www.ncbi.nlm.nih.gov/pubmed/15960930/) |
|  |  |  |  | Risk Factors for Early-Onset and Very-Early-Onset Pancreatic Adenocarcinoma | | 2016 | [https://journals.lww.com/pancreasj](https://journals.lww.com/pancreasjournal/Abstract/2016/02000/Risk_Factors_for_Early_Onset_and_Very_Early_Onset.23.aspx)ournal/Abstract/2016/02000/Risk_[Factors_for_Early_Onset_and_Very_Early_Onset.23.aspx](https://journals.lww.com/pancreasjournal/Abstract/2016/02000/Risk_Factors_for_Early_Onset_and_Very_Early_Onset.23.aspx) |
|  |  |  |  | Non-steroidal anti-inflammatory  drugs and pancreatic cancer risk: a nested case–control study | | 2012 | <https://www.ncbi.nlm.nih.gov/pmc/articles/PMC2865751/> |
|  |  |  |  | Impact of diabetes duration and chronic pancreatitis on the association between type 2  diabetes and pancreatic | | 2012 | [https://www.ncbi.nlm.nih.gov/pubmed/22831166](https://www.ncbi.nlm.nih.gov/pubmed/15960930/) |
| Anaemia | 207 | *anaemia*, *anaemi*,  *anaemic*, *anemi* |  |  | |  |  |
| Abdominal pain | 78 |  | https://bmjopen.bmj.com/content/6/5/e011664.full appendix 2 | Signs and symptoms of pancreatic  cancer: a population-based case-  control study in the San Francisco Bay area. | | 2016 | <https://www.ncbi.nlm.nih.gov/pubmed/15181621/> |
|  |  |  |  | Exocrine pancreatic cancer: symptoms at presentation and their  relation to tumour site and stage | | 2005 | <https://www.ncbi.nlm.nih.gov/pubmed/15960930/> |
|  |  |  |  | The risk of pancreatic cancer in symptomatic patients in primary care: a large case-control study  using electronic records | | 2012 | <https://www.ncbi.nlm.nih.gov/pubmed/22617126> |
| Diabetes [including patients on  anti- diabetics(metformin,sulfonylureas, | 324 | *diabetes* *mellitus*,  *diabetes* |  | Recent Progress in Pancreatic  Cancer | | 2013 | [https://www.ncbi.nlm.nih.gov/pmc/articles/PMC3769458/](https://www.ncbi.nlm.nih.gov/pubmed/15960930/) |
|  | 517 |  | [https://datacompass.lshtm.ac.uk/1122/ - Bhaskaran,](https://datacompass.lshtm.ac.uk/1122/%20-%20Bhaskaran%2C%20K) [K](https://datacompass.lshtm.ac.uk/1122/%20-%20Bhaskaran%2C%20K) | The risk of pancreatic cancer in symptomatic patients in primary care: a large case-control study  using electronic records | | 2012 | <https://www.ncbi.nlm.nih.gov/pubmed/22617126> |
|  | 689 |  | https://datacompass.lshtm.ac.uk/1231/1/Antidiabeti cs_CPRD.txt | Non-steroidal anti-inflammatory drugs and pancreatic cancer risk: a  nested case–control study | | 2012 | <https://www.ncbi.nlm.nih.gov/pmc/articles/PMC2865751/> |
|  | 78 | *metformin* |  | Risk Factors for Pancreatic  Cancer: Case-Control Study | | 2007 | <https://www.ncbi.nlm.nih.gov/pmc/articles/PMC2423805/> |
|  | 304 product codes from code browser | *insulin* |  | Risk Factors for Early-Onset and Very-Early-Onset Pancreatic Adenocarcinoma | | 2016 | <https://journals.lww.com/pancreasjournal/Abstract/2016/02000/Risk_Factors_for_Early_Onset_and_Very_Early_Onset.23.aspx> |
|  | 0 | *sulfonylureas*,  *sulfonylur*, *sulfony*,  *anti* *diabetic*,  *sulfon* *lureas* |  | Impact of diabetes duration and chronic pancreatitis on the association between type 2  diabetes and pancreatic | | 2012 | [https://www.ncbi.nlm.nih.gov/pubmed/22831166](https://www.ncbi.nlm.nih.gov/pubmed/15960930/) |
|  |  |  |  | An Absolute Risk Model to Identify Individuals at Elevated Risk for Pancreatic Cancer in the  General Population | | 2013 | [https://www.ncbi.nlm.nih.gov/pmc/articles/PMC3772857/#](https://www.ncbi.nlm.nih.gov/pubmed/15960930/) |
|  |  |  |  | Use of antidiabetic agents and the  risk of pancreatic cancer: a case- control analysis. | | 2012 | <https://www.ncbi.nlm.nih.gov/pubmed/22290402> |
| Constipation | 11 | *constipation*,  *constipa* |  | Signs and symptoms of pancreatic  cancer: a population-based case- control study in the San Francisco Bay area. | | 2016 | <https://www.ncbi.nlm.nih.gov/pubmed/15181621/> |
|  |  |  |  | The risk of pancreatic cancer in symptomatic patients in primary care: a large case-control study  using electronic records | | 2012 | <https://www.ncbi.nlm.nih.gov/pubmed/22617126> |
| Jaundice [including Cholestatic  symptoms] | 52 | *jaundice* |  | Recent Progress in Pancreatic  Cancer | | 2013 | [https://www.ncbi.nlm.nih.gov/pmc/articles/PMC3769458/](https://www.ncbi.nlm.nih.gov/pubmed/15960930/) |
|  | 46 | *Cholestatic*, Cholestat*,  *cholestasis*,  *cholangitis*,*cholecystit is* |  | The risk of pancreatic cancer in symptomatic patients in primary care: a large case-control study  using electronic records | | 2012 | <https://www.ncbi.nlm.nih.gov/pubmed/22617126> |
|  |  |  |  | Signs and symptoms of pancreatic cancer: a population-based case- control study in the San Francisco  Bay area. | | 2016 | <https://www.ncbi.nlm.nih.gov/pubmed/15181621/> |
|  |  |  |  | Symptoms of Pancreatic Cancer in Primary Care | | 2016 | [https://journals.lww.com/pancreasjournal/Abstract/2016/07000/Symptoms_of_Pancreatic_Cancer_in_Primary_Care A.7.aspx](https://journals.lww.com/pancreasjournal/Abstract/2016/07000/Symptoms_of_Pancreatic_Cancer_in_Primary_Care__A.7.aspx) |
|  |  |  |  | Exocrine pancreatic cancer:  symptoms at presentation and their relation to tumour site and stage | | 2005 | <https://www.ncbi.nlm.nih.gov/pubmed/15960930/> |
| Oesophago-gastric problems | 342 | *oesophago* ,  *oesophago* *gastric*,  *gastric*, *oesophago- gastric* , *oesophago gastric* , *oesopha* |  |  | |  |  |
| Back pain [epigastric pain that  radiates to the back] | 13 | *back* *pain* |  | Recent Progress in Pancreatic  Cancer | | 2013 | [https://www.ncbi.nlm.nih.gov/pmc/articles/PMC3769458/](https://www.ncbi.nlm.nih.gov/pubmed/15960930/) |
|  |  |  |  | The risk of pancreatic cancer in symptomatic patients in primary care: a large case-control study  using electronic records | | 2012 | <https://www.ncbi.nlm.nih.gov/pubmed/22617126> |
| Gastrointestinal conditions  [including diarrhoea, nausea/ vomiting] | 37 | *heartburn*,  *indigestion*, *peptic*  *ulcer*, *feel* *full*,  *satiety*, *gastroparesis*, |  | Signs and symptoms of pancreatic  cancer: a population-based case- control study in the San Francisco Bay area. | | 2004 | <https://www.ncbi.nlm.nih.gov/pubmed/15181621/> |
|  | 18 | *diarrhoea* | [https://datacompass.lshtm.ac.uk/1193/1/rota_diag_c](https://datacompass.lshtm.ac.uk/1193/1/rota_diag_codelist_hf.txt) [odelist_hf.txt](https://datacompass.lshtm.ac.uk/1193/1/rota_diag_codelist_hf.txt) | Signs and symptoms of pancreatic  cancer: a population-based case- control study in the San Francisco Bay area. | | 2016 | <https://www.ncbi.nlm.nih.gov/pubmed/15181621/> |
|  | 151 |  | [https://datacompass.lshtm.ac.uk/522/1/medcodes-](https://datacompass.lshtm.ac.uk/522/1/medcodes-GE-sccs.txt) [GE-sccs.txt](https://datacompass.lshtm.ac.uk/522/1/medcodes-GE-sccs.txt) | The risk of pancreatic cancer in symptomatic patients in primary care: a large case-control study  using electronic records | | 2012 | <https://www.ncbi.nlm.nih.gov/pubmed/22617126> |
|  | 70 | *nausea*, *vomiting*,  *vomit*, *sick*, |  | Recent Progress in Pancreatic  Cancer | | 2013 | <https://www.ncbi.nlm.nih.gov/pmc/articles/PMC3769458/> |
|  | 30 |  | [https://datacompass.lshtm.ac.uk/851/1/Clinical_cod](https://datacompass.lshtm.ac.uk/851/1/Clinical_codelist_ICD-10_GIbleeding.txt) [elist_ICD-](https://datacompass.lshtm.ac.uk/851/1/Clinical_codelist_ICD-10_GIbleeding.txt) [10_GIbleeding.txt](https://datacompass.lshtm.ac.uk/851/1/Clinical_codelist_ICD-10_GIbleeding.txt) | The risk of pancreatic cancer in symptomatic patients in primary care: a large case-control study  using electronic records | | 2012 | <https://www.ncbi.nlm.nih.gov/pubmed/22617126> |
|  | 61 |  | [https://datacompass.lshtm.ac.uk/1193/1/rota_diag_c](https://datacompass.lshtm.ac.uk/1193/1/rota_diag_codelist_hf.txt) |  | |  |  |
| Irritable bowel syndrome | 9 | *irr* *bowel*,*change*  *bowel*, *abnormal*  *bowel* *sound*, *IBS*, |  |  | |  |  |
| Diverticular disease | 6 | *diverticu* *dis* |  |  | |  |  |
| Autoimmune conditions [Coeliac  disease, AI haemolytic anaemias, Allergic purpura, ITP, MS, AI hepatitis,Pemphigus,Psoriasis,conn ective tissue disease, Lupus, Sarcoidosis, Graves' disease, Hashimoto thyroiditis, Primary biliary cirrhosis] | 167 | *coelia* *dis*,  *autoimm* *haemolytic*  *anaemia*, *aller*  *purpur*, *itp*, *idio*  *throm* *purpur*,  *multi* *scler*, *autoi*  *hepa*, *ai*  *hepa*,*pemphi*,  *psorias*, *connec* |  |  | |  |  |
|  | 28 |  | [.ac.uk/1132/1/cr_codelist](https://datacompass.lshtm.ac.uk/1132/1/cr_codelist_sle.csv)  [_sle.csv](https://datacompass.lshtm.ac.uk/1132/1/cr_codelist_sle.csv) |  | |  |  |
| Inflammatory bowel diseases | 45 |  | [https://datacompass.lshtm.ac.uk/347/1/IBD_Codes.t](https://datacompass.lshtm.ac.uk/347/1/IBD_Codes.txt) |  | |  |  |
| Gynaecological conditions | 69 | *gynae* |  |  | |  |  |
| Endometriosis | 92 | *endometr* |  |  | |  |  |
| Dysmenorrhoea | 7 | *dysmen* |  |  | |  |  |
| Fibroids | 24 | *fibroi* |  |  | |  |  |
| Rheumatoid arthritis | 58 | *rheumatoid* *arthritis*,  *Rheumatoid arthritis* |  | Non-steroidal anti-inflammatory  drugs and pancreatic cancer risk: a nested case–control study | | 2010 | <https://www.ncbi.nlm.nih.gov/pmc/articles/PMC2865751/> |
|  | 56 |  | https://datacompass.lshtm.ac.uk/355/1/Rheumatoid |  | |  |  |
| Gallbladder disease | 60 | *gallbladder* *dis*,  *gallbladder* *mal*,  *gallbladder* |  | Impact of diabetes duration and  chronic pancreatitis on the association between type 2 diabetes and pancreatic | | 2012 | [https://www.ncbi.nlm.nih.gov/pubmed/22831166](https://www.ncbi.nlm.nih.gov/pubmed/15960930/) |
| Choluria | 0 | *choluria*, *bile* *urine* |  | Exocrine pancreatic cancer:  symptoms at presentation and their relation to tumour site and stage | | 2005 | <https://www.ncbi.nlm.nih.gov/pubmed/15960930/> |
| Migratory thrombophlebitis  (Trousseau’s syndrome) | 1 | *thrombophlebitis*,  *thrombophlebit*, *mig*  *thrombophlebit* ,  *thrombophlebit* *mig*,  *trousseau* |  | Recent Progress in Pancreatic  Cancer | | 2013 | <https://www.ncbi.nlm.nih.gov/pmc/articles/PMC3769458/> |
| Cardiovascular disease [including  DVT/PE and Arrhythmia] | 50 | *dvt*, *deep* *vein*  thr*, *pulmo* *embo* |  |  | |  |  |
|  | 904 |  | [.ac.uk/1116/1/cr_codelist](https://datacompass.lshtm.ac.uk/1116/1/cr_codelist_cvd.csv)  [_cvd.csv](https://datacompass.lshtm.ac.uk/1116/1/cr_codelist_cvd.csv) |  | |  |  |
|  | 17 |  | [https://datacompass.lshtm.ac.uk/1475/1/codelist-](https://datacompass.lshtm.ac.uk/1475/1/codelist-medcodes-arrhythmia-aki2.txt) [medcodes-arrhythmia-](https://datacompass.lshtm.ac.uk/1475/1/codelist-medcodes-arrhythmia-aki2.txt) |  | |  |  |
| Hypertension | 127 |  | [.ac.uk/1082/1/audit_hypertension.txt](https://datacompass.lshtm.ac.uk/1082/1/audit_hypertension.txt) |  | |  |  |
| Atopic diseases [Asthma,  Eczema,Allergic rhinitis, contact dermatitis] | 63 | *eczema*, *rhiniti*,  *dermatitis* |  |  | |  |  |
|  | 137 |  | [https://datacompass.lshtm](https://datacompass.lshtm.ac.uk/829/1/cr_asthma_hf.txt)[.ac.uk/829/1/cr_asthma_h](https://datacompass.lshtm.ac.uk/718/1/CKD-codes.txt) |  | |  |  |
| Kidney diseases | 24 |  | [.ac.uk/718/1/CKD-](https://datacompass.lshtm.ac.uk/718/1/CKD-codes.txt)  [codes.txt](https://datacompass.lshtm.ac.uk/718/1/CKD-codes.txt) |  | |  |  |
|  | 37 | *pyelonephritis*, |  |  | |  |  |
| Urinary diseases | 25 | *polyuria*,*biluria*,*dar  k* *urine*,*dark* *urin*,  *hematuria*, |  |  | |  |  |
|  | 20 |  | [.ac.uk/470/1/Clinical_cod](https://datacompass.lshtm.ac.uk/470/1/Clinical_codelist_UTI.txt)  [elist_UTI.txt](https://datacompass.lshtm.ac.uk/470/1/Clinical_codelist_UTI.txt) |  | |  |  |
| Hypersomnia | 7 | *hypersomnia*,  *sleepy*,*sleep*  *day*,*excess* *sleep* |  |  | |  |  |
| Insomnia | 25 | *insomnia*, *sleepless*,  *sleep* *dis* |  |  | |  |  |
| Glossodynia | 2 | *glossodynia* |  |  | |  |  |
| Halitosis | 7 | *bad* *breath*,  *halitosis*, *off* |  |  | |  |  |
| Xerostomia | 4 | *xerostomia*, *dry*  *mouth*, *dry* *saliva* |  |  | |  |  |
| Steatorrhoea | 2 | *steatorrhoea*, *fat*  *feces*, *fat* *feca* |  |  | |  |  |
| Flatulence | 11 | *flatul*, *wind* |  |  | |  |  |
| Pruritis/Itching | 8 | *pruritis*,*itchi*, |  |  | |  |  |
| Abdominal mass | 10 | *abdo* *mass*,*abdo* |  |  | |  |  |
| Polydipsia | 7 | *polydipsia*, *excess* |  |  | |  |  |
| Odynophagia/ Dysphagia | 9 | *pain* *swallow*,  *odynophagia*, |  |  | |  |  |
| Oral diseases [Caries, Pulp and  periapical tissue disease, gingivitis/peridontal disease] | 48 | *caries*, *pulp* *peria*  *tiss* ,*peria* *tiss* ,  *pulp* *peria* ,  *periapic* ,*gingivit*, |  |  | |  |  |
| Mumps | 22 | *mump* |  |  | |  |  |
| Stomatitis [including cheilitis] | 27 | *stomatitis*, *cheilitis*,  *inflam* *muco* |  |  | |  |  |
| Hyperlipidaemia/Hypercholesterol  aemia | 55 |  | [.ac.uk/834/1/cr_hyperlipi](https://datacompass.lshtm.ac.uk/834/1/cr_hyperlipidemia_hf.txt)  [demia_hf.txt](https://datacompass.lshtm.ac.uk/834/1/cr_hyperlipidemia_hf.txt) |  | |  |  |
|  | 1 | *hypercholester*,  *hypertriglyceridaemia*,  *dyslipidaemia* |  |  | |  |  |
| Histamine-2-receptor antagonists  (H2RAs) | 0 | *histamine* *recep*,  *hist* *recep* |  | Proton pump inhibitors and  histamine-2-receptor antagonists and pancreatic cancer risk: a nested case–control study | | 2011 | <https://www.ncbi.nlm.nih.gov/pmc/articles/PMC3251858/> |
|  |  |  |  | Non-steroidal anti-inflammatory drugs and pancreatic cancer risk: a  nested case–control study | | 2012 | <https://www.ncbi.nlm.nih.gov/pmc/articles/PMC2865751/> |
| Proton pump inhibitors (PPI) | 0 | *prot* *pump* *inhi*,  *inhibitor*, *proton* |  | Proton pump inhibitors and  histamine-2-receptor antagonists and pancreatic cancer risk: a  nested case–control study | | 2011 | <https://www.ncbi.nlm.nih.gov/pmc/articles/PMC3251858/> |
|  |  |  |  | Non-steroidal anti-inflammatory  drugs and pancreatic cancer risk: a nested case–control study | | 2012 | <https://www.ncbi.nlm.nih.gov/pmc/articles/PMC2865751/> |
|  |  |  |  | Proton pump inhibitors on  pancreatic cancer risk and survival | | 2017 | <https://www.ncbi.nlm.nih.gov/pubmed/28056391/> |
|  |  |  |  | Proton Pump Inhibitor Use is Associated With Risk of Pancreatic Cancer: A Nested Case-  Control Study | | 2018 | <https://www.ncbi.nlm.nih.gov/pubmed/30288155> |
| Systemic steroids | 33 | *systemic* *steroids*,  *sys* *steroids*, *sys*  *steroid*, *sys* *ster*,  *corticosteroid*,  *cortisone*,  *prednisone*, |  | Non-steroidal anti-inflammatory  drugs and pancreatic cancer risk: a nested case–control study | | 2010 | <https://www.ncbi.nlm.nih.gov/pmc/articles/PMC2865751/> |
| Hormone replacement therapy  (HRT) | 11 | *hormone* *rep* *ther* |  | Non-steroidal anti-inflammatory  drugs and pancreatic cancer risk: a nested case–control study | | 2011 | <https://www.ncbi.nlm.nih.gov/pmc/articles/PMC2865751/> |
|  | 282 |  | https://datacompass.lshtm.ac.uk/1129/1/cr_codelist |  | |  |  |
| Disease modifying anti-rheumatic  drugs | 0 | *Disease modifying anti-  rheumatic drugs*, *anti- rheumatic drugs*,  *antirheumatic drugs*, |  | Non-steroidal anti-inflammatory  drugs and pancreatic cancer risk: a nested case–control study | | 2012 | <https://www.ncbi.nlm.nih.gov/pmc/articles/PMC2865751/> |
| ABO genotype, 1q32 rs3790844,  5p15 rs401681, 13q22 rs9543325 | 0 | *abo* *genotype*,  *genotype* |  | Recent Progress in Pancreatic  Cancer | | 2013 | <https://www.ncbi.nlm.nih.gov/pmc/articles/PMC3769458/> |
|  |  |  |  | An Absolute Risk Model to Identify Individuals at Elevated Risk for Pancreatic Cancer in the  General Population | | 2013 | [https://www.ncbi.nlm.nih.gov/pmc/articles/PMC3772857/#](https://www.ncbi.nlm.nih.gov/pmc/articles/PMC3772857/) |
| Nonsteroidal anti-inflammatory  drugs (NSAIDs) or aspirin | 1191 |  | [https://datacompass.lshtm.ac.uk/1128/1/cr_codelist](https://datacompass.lshtm.ac.uk/1128/1/cr_codelist_nsaids.csv)_[nsaids.csv](https://datacompass.lshtm.ac.uk/1128/1/cr_codelist_nsaids.csv) | Non-steroidal anti-inflammatory  drugs and pancreatic cancer risk: a nested case–control study | | 2010 | <https://www.ncbi.nlm.nih.gov/pmc/articles/PMC2865751/> |
|  |  |  |  | Proton pump inhibitors and histamine-2-receptor antagonists and pancreatic cancer risk: a  nested case–control study | | 2011 | <https://www.ncbi.nlm.nih.gov/pmc/articles/PMC3251858/> |
| Opioids | 957 |  | [.ac.uk/1424/1/cr_codelist](https://datacompass.lshtm.ac.uk/1424/1/cr_codelist_opioids.txt)  [_opioids.txt](https://datacompass.lshtm.ac.uk/1424/1/cr_codelist_opioids.txt) |  | |  |  |
| Antiplatelets | 271 |  | [https://datacompass.lshtm](https://datacompass.lshtm.ac.uk/892/1/Therapy_codelist_antiplatelets.csv)  [.ac.uk/892/1/Therapy_cod](https://datacompass.lshtm.ac.uk/892/1/Therapy_codelist_antiplatelets.csv) [elist_antiplatelets.csv](https://datacompass.lshtm.ac.uk/892/1/Therapy_codelist_antiplatelets.csv) |  | |  |  |
| Primary care consulation  frequency | calculated using CPRD  Consultation file |  |  |  | |  |  |
