# Supplementary material for: Can we screen for pancreatic cancer? Identifying a sub-population of patients at high risk of subsequent diagnosis using machine learning techniques applied to primary care data
Source: PLoS One. 2021 Jun 2;16(6):e0251876. doi: 10.1371/journal.pone.0251876 (PMC8171946; doi:10.1371/journal.pone.0251876)
Supplement: S2 Table — (DOCX) [file pone.0251876.s012.docx]

**S2 Table. Multivariate logistic regression model fitted at month 20 before diagnosis for age-group up to 60 years.**

|  | **Estimate** | **Standard Error** | ***p*-value** | **Odds Ratio** |
| --- | --- | --- | --- | --- |
| (Intercept) | -0.65 | 0.32 | 0.04 | 0.52 |
| Sex[male] | -0.03 | 0.24 | 0.91 | 0.97 |
| Consultation Frequency | -0.05 | 0.06 | 0.38 | 0.95 |
| Deprivation | -0.10 | 0.09 | 0.25 | 0.90 |
| Ever smoker | 0.27 | 0.25 | 0.27 | 1.31 |
| Ever heavy drinker | 0.61 | 0.95 | 0.52 | 1.85 |
| Diabetes | 1.12 | 0.37 | 0.00 | 3.06 |
| Obesity | 0.28 | 0.34 | 0.40 | 1.33 |
| Weight loss | -1.53 | 1.18 | 0.20 | 0.22 |
| NSAIDS | -0.09 | 0.28 | 0.75 | 0.91 |
| Opioids | 0.22 | 0.15 | 0.15 | 1.24 |
| Antiplatelets | 0.05 | 0.20 | 0.80 | 1.05 |
| HRT | 0.35 | 0.28 | 0.21 | 1.42 |
| Jaundice | 17.51 | >100 | 0.99 | >100 |
| Abdominal pain | 0.83 | 0.69 | 0.23 | 2.29 |
| Anaemia | -5.48 | >100 | 0.99 | 0.00 |
| Anxiety/Depression | -0.09 | 0.66 | 0.90 | 0.92 |
| Weakness | -16.45 | >100 | 0.99 | 0.00 |
| Back pain | -0.29 | 0.56 | 0.61 | 0.75 |
| Gastrointestinal conditions | 0.28 | 0.50 | 0.58 | 1.32 |
| Rheumatoid arthritis | -7.83 | >100 | 0.99 | 0.00 |
| Fatigue/Malaise | -16.29 | >100 | 0.99 | 0.00 |
| Irritable bowel syndrome | 0.02 | 0.97 | 0.98 | 1.02 |
| Gynaecological conditions | -16.37 | >100 | 0.99 | 0.00 |
| Gallbladder diseases | -15.88 | >100 | 0.99 | 0.00 |
| Oesophago-gastric problems | 0.65 | 1.44 | 0.65 | 1.92 |
| Cardiovascular diseases | 0.15 | 0.43 | 0.72 | 1.17 |
| Hypertension | 0.10 | 0.39 | 0.79 | 1.11 |
| Atopic diseases | 0.30 | 0.43 | 0.48 | 1.36 |
| Kidney problems | -15.23 | >100 | 0.99 | 0.00 |
| Other urinary problems | -0.86 | 1.30 | 0.51 | 0.42 |
| Insomnia | 1.47 | 1.32 | 0.27 | 4.33 |
| Fever | 1.00 | 1.36 | 0.46 | 2.71 |
| Auto-immune diseases | 0.34 | 1.11 | 0.76 | 1.40 |
| Hyperlipidaemia | 0.00 | 1.01 | 1.00 | 1.00 |
