# Supplementary material for: Can we screen for pancreatic cancer? Identifying a sub-population of patients at high risk of subsequent diagnosis using machine learning techniques applied to primary care data
Source: PLoS One. 2021 Jun 2;16(6):e0251876. doi: 10.1371/journal.pone.0251876 (PMC8171946; doi:10.1371/journal.pone.0251876)
Supplement: S3 Table — (DOCX) [file pone.0251876.s013.docx]

**S3 Table.** **Multivariate logistic regression model fitted at month 17 before diagnosis for age-group above 60 years.**

|  | **Estimate** | **Standard Error** | ***p*-value** | **Odds Ratio** |
| --- | --- | --- | --- | --- |
| (Intercept) | -1.45 | 0.14 | 0.00 | 0.23 |
| Sex [male] | -0.15 | 0.10 | 0.15 | 0.86 |
| Consultation frequency | 0.00 | 0.01 | 0.99 | 1.00 |
| Deprivation | -0.04 | 0.04 | 0.37 | 0.97 |
| Ever smoker | 0.52 | 0.10 | < 0.001 | 1.68 |
| Ever heavy drinker | 0.38 | 0.51 | 0.46 | 1.46 |
| Diabetes | 0.75 | 0.13 | 0.00 | 2.11 |
| Obesity | -0.02 | 0.14 | 0.87 | 0.98 |
| Weight loss | 0.38 | 0.28 | 0.18 | 1.46 |
| NSAIDS | 0.02 | 0.04 | 0.61 | 1.02 |
| Opioids | 0.08 | 0.04 | 0.04 | 1.09 |
| Antiplatelets | 0.03 | 0.02 | 0.07 | 1.03 |
| HRT | -0.18 | 0.16 | 0.25 | 0.84 |
| Jaundice | 15.24 | >100 | 0.98 | >100 |
| Abdominal pain | 0.26 | 0.20 | 0.20 | 1.30 |
| Abdominal mass | -7.41 | >100 | 0.99 | 0.00 |
| Anaemia | -0.11 | 0.40 | 0.78 | 0.89 |
| Anorexia | 0.36 | 0.62 | 0.56 | 1.44 |
| Anxiety/Depression | -0.23 | 0.24 | 0.34 | 0.79 |
| Weakness | 0.09 | 0.52 | 0.87 | 1.09 |
| Back pain | 0.07 | 0.18 | 0.72 | 1.07 |
| Gastrointestinal conditions | 0.03 | 0.10 | 0.77 | 1.03 |
| Rheumatoid arthritis | 0.60 | 0.69 | 0.39 | 1.82 |
| Fatigue/Malaise | 0.27 | 0.29 | 0.35 | 1.31 |
| Irritable bowel syndrome | -0.14 | 0.48 | 0.77 | 0.87 |
| Inflammatory bowel disease | -14.70 | >100 | 0.99 | 0.00 |
| Diverticular disease | -0.22 | 0.59 | 0.71 | 0.80 |
| Gynaecological conditions | -0.14 | 0.34 | 0.69 | 0.87 |
| Endometriosis | -13.90 | >100 | 0.99 | 0.00 |
| Gallbladder disease | -0.96 | 1.17 | 0.41 | 0.38 |
| Constipation | 0.34 | 0.26 | 0.20 | 1.40 |
| Oesophago-gastric problems | -0.62 | 0.44 | 0.16 | 0.54 |
| Cardiovascular diseases | 0.00 | 0.07 | 0.95 | 1.00 |
| Hypertension | 0.02 | 0.09 | 0.86 | 1.02 |
| Atopic diseases | 0.06 | 0.07 | 0.41 | 1.06 |
| Kidney problems | -0.34 | 0.38 | 0.36 | 0.71 |
| Other urinary problems | 0.07 | 0.15 | 0.62 | 1.08 |
| Insomnia | 0.54 | 0.42 | 0.20 | 1.72 |
| Stomatitis | -14.85 | >100 | 0.99 | 0.00 |
| Xerostomia | -14.99 | >100 | 0.99 | 0.00 |
| Flatulence | -14.39 | >100 | 0.99 | 0.00 |
| Pruritis | -0.32 | 0.56 | 0.57 | 0.73 |
| Polydipsia | -14.66 | >100 | 0.99 | 0.00 |
| Fever | -0.38 | 0.71 | 0.60 | 0.68 |
| Odynophagia | 2.56 | 1.21 | 0.03 | 13.00 |
| Auto-immune diseases | 0.17 | 0.21 | 0.41 | 1.19 |
| Mumps | 16.69 | >100 | 0.99 | >100 |
| Hyperlipidaemia | -0.02 | 0.28 | 0.95 | 0.98 |
