# Supplementary material for: Can we screen for pancreatic cancer? Identifying a sub-population of patients at high risk of subsequent diagnosis using machine learning techniques applied to primary care data
Source: PLoS One. 2021 Jun 2;16(6):e0251876. doi: 10.1371/journal.pone.0251876 (PMC8171946; doi:10.1371/journal.pone.0251876)
Supplement: S4 Table — (DOCX) [file pone.0251876.s014.docx]

**S4 Table. Random forest model fitted at month 20 before diagnosis for age-group up to 60 years**

|  | **Mean decrease in**  **Accuracy** | **Mean decrease in**  **Gini score** |
| --- | --- | --- |
| Diabetes | 22.09 | 2.81 |
| Opioids | 11.08 | 1.55 |
| Weight loss | 7.09 | 0.70 |
| Consultation frequency | 3.08 | 5.91 |
| Fatigue/Malaise | 2.45 | 0.09 |
| Cardiovascular diseases | 0.79 | 0.78 |
| Antiplatelets | 0.23 | 1.16 |
| Atopic diseases | 0.21 | 0.94 |
| Ever smoker | 0.15 | 1.65 |
| Jaundice | 0.00 | 0.18 |
| Anaemia | 0.00 | 0.03 |
| Weakness | 0.00 | 0.06 |
| Rheumatoid arthritis | 0.00 | 0.05 |
| Gynaecological conditions | 0.00 | 0.03 |
| Gallbladder disease | 0.00 | 0.05 |
| Kidney problems | 0.00 | 0.01 |
| Obesity | -0.14 | 1.40 |
| Insomnia | -0.17 | 0.48 |
| Ever heavy drinker | -1.47 | 0.59 |
| Back pain | -1.65 | 0.72 |
| Abdominal pain | -1.72 | 0.43 |
| Irritable bowel syndrome | -2.20 | 0.30 |
| HRT | -2.55 | 1.35 |
| Deprivation | -2.63 | 4.28 |
| Other urinary problems | -3.10 | 0.26 |
| NSAIDs | -3.15 | 1.51 |
| Auto-immune diseases | -3.22 | 0.36 |
| Fever | -3.70 | 0.29 |
| Oesophago-gastric problems | -3.92 | 0.23 |
| Sex [male] | -4.03 | 1.61 |
| Hypertension | -4.47 | 1.02 |
| Gastrointestinal conditions | -5.73 | 0.86 |
| Hyperlipidaemia | -5.89 | 0.40 |
| Anxiety/Depression | -6.18 | 0.64 |
