# Supplementary material for: Can we screen for pancreatic cancer? Identifying a sub-population of patients at high risk of subsequent diagnosis using machine learning techniques applied to primary care data
Source: PLoS One. 2021 Jun 2;16(6):e0251876. doi: 10.1371/journal.pone.0251876 (PMC8171946; doi:10.1371/journal.pone.0251876)
Supplement: S5 Table — (DOCX) [file pone.0251876.s015.docx]

**S5 Table. Random forest model fitted at month 17 before diagnosis for age-group above 60 years.**

|  | **Mean decrease in**  **Accuracy** | **Mean decrease in**  **Gini score** |
| --- | --- | --- |
| Diabetes | 31.55 | 11.41 |
| Ever smoker | 22.25 | 7.92 |
| Opioids | 17.22 | 8.17 |
| Antiplatelets | 15.68 | 17.77 |
| Consultation frequency | 12.34 | 28.33 |
| Other urinary problems | 11.39 | 4.58 |
| Cardiovascular diseases | 9.77 | 6.79 |
| Weight loss | 9.38 | 3.12 |
| Fatigue/Malaise | 8.78 | 2.77 |
| Gastrointestinal conditions | 7.83 | 5.49 |
| Jaundice | 6.78 | 0.92 |
| Ever heavy drinker | 5.73 | 1.83 |
| Weakness | 5.68 | 1.26 |
| Anaemia | 5.32 | 1.33 |
| Sex [male] | 5.30 | 6.13 |
| Hypertension | 5.07 | 7.99 |
| Abdominal pain | 4.08 | 3.67 |
| Anorexia | 3.91 | 0.52 |
| Insomnia | 3.89 | 2.15 |
| Diverticular disease | 3.89 | 0.77 |
| Deprivation | 3.16 | 16.22 |
| Odynophagia | 2.82 | 1.01 |
| Polydipsia | 2.00 | 0.09 |
| Back pain | 1.73 | 3.77 |
| Inflammatory bowel disease | 1.61 | 0.08 |
| Hyperlipidaemia | 0.61 | 2.55 |
| NSAIDs | 0.12 | 10.57 |
| Abdominal mass | 0.00 | 0.03 |
| Endometriosis | 0.00 | 0.01 |
| Flatulence | 0.00 | 0.01 |
| Mumps | 0.00 | 0.51 |
| Gallbladder disease | -0.31 | 0.40 |
| Obesity | -0.33 | 4.86 |
| HRT | -0.71 | 2.99 |
| Xerostomia | -1.00 | 0.05 |
| Stomatitis | -1.08 | 0.02 |
| Fever | -1.08 | 0.53 |
| Gynaecological conditions | -1.10 | 1.61 |
| Atopic diseases | -1.31 | 5.83 |
| Constipation | -1.45 | 2.08 |
| Rheumatoid arthritis | -2.37 | 0.80 |
| Irritable bowel syndrome | -2.77 | 1.04 |
| Pruritis | -4.68 | 0.81 |
| Kidney problems | -4.89 | 1.50 |
| Oesophago-gastric problems | -5.31 | 1.16 |
| Auto-immune diseases | -5.72 | 2.30 |
| Anxiety/Depression | -7.76 | 2.88 |
